# Ethylene –dependent and –independent superficial scald resistance mechanisms in ‘Granny Smith’ apple fruit

**DOI:** 10.1038/s41598-018-29706-x

**Published:** 2018-07-30

**Authors:** Evangelos Karagiannis, Michail Michailidis, Georgia Tanou, Martina Samiotaki, Katerina Karamanoli, Evangelia Avramidou, Ioannis Ganopoulos, Panagiotis Madesis, Athanassios Molassiotis

**Affiliations:** 1 Laboratory of Pomology, Department of Agriculture, Aristotle University of Thessaloniki, 54124 Thessaloniki, Greece; 2 Institute of Soil and Water Resources, ELGO-DEMETER, Thermi, Thessaloniki 57001 Greece; 3 Biomedical Sciences Research Center “Alexander Fleming”, Vari, 16672 Greece; 4 Laboratory of Agricultural Chemistry, Department of Agriculture, Aristotle University of Thessaloniki, 54124 Thessaloniki, Greece; 5 Laboratory of Forest Genetics and Biotechnology, Institute of Mediterranean Forest Ecosystems, Athens, 11528 Greece; 6 Institute of Plant Breeding and Genetic Resources, ELGO-DEMETER, Thermi, Thessaloniki Greece; 7 Institute of Applied Biotechnology, CERTH, Thessaloniki, Greece

## Abstract

Superficial scald is a major physiological disorder of apple fruit (*Malus domestica* Borkh.) characterized by skin browning following cold storage; however, knowledge regarding the downstream processes that modulate scald phenomenon is unclear. To gain insight into the mechanisms underlying scald resistance, ‘Granny Smith’ apples after harvest were treated with diphenylamine (DPA) or 1-methylcyclopropene (1-MCP), then cold stored (0 °C for 3 months) and subsequently were ripened at room temperature (20 °C for 8 days). Phenotypic and physiological data indicated that both chemical treatments induced scald resistance while 1-MCP inhibited the ethylene-dependent ripening. A combination of multi-omic analysis in apple skin tissue enabled characterization of potential genes, proteins and metabolites that were regulated by DPA and 1-MCP at pro-symptomatic and scald-symptomatic period. Specifically, we characterized strata of scald resistance responses, among which we focus on selected pathways including dehydroabietic acid biosynthesis and UDP-D-glucose regulation. Through this approach, we revealed scald-associated transcriptional, proteomic and metabolic signatures and identified pathways modulated by the common or distinct functions of DPA and 1-MCP. Also, evidence is presented supporting that cytosine methylation-based epigenetic regulation is involved in scald resistance. Results allow a greater comprehension of the ethylene–dependent and –independent metabolic events controlling scald resistance.

## Introduction

Cold stress is a major abiotic challenge that limits the crop production both in the field and after harvesting^1^. Fruits, like apples, are artificially subjected to cold storage to delay ripening and senescence^2^. However, prolonged cold exposure of apples provoke stress conditions leading to cellular disruption and superficial scald expression, a major physiological disorder that is characterized by skin browning limited to the skin and the underlying cell layers^3,4^. Scald symptoms mainly occur during ripening at room temperature (20 °C) following cold exposure (−1 to 4 °C)^3^. Despite its agronomic and scientific importance, the mechanisms that stimulate or limit scald development have remained an enigma^4^.

A long-standing hypothesis holds that scald symptoms are caused by oxidative reactions, resulting in the production of conjugated trienol oxidative products of the sesquiterpene *α*-farnesene, which accumulate during storage in response to cold stress. The synthesis of *α*-farnesene is closely linked to ethylene production^5^, since this hormone modulates the expression of the gene MdAFS1 encoding *α*-farnesene synthase 1, the last enzyme in the *α*-farnesene biosynthetic pathway. Most research effort to date on scald etiology has been dedicated to *α*-farnesene oxidation while alternative ideas remained relatively poorly defined^6^. Recent analysis in ‘Granny Smith’ apples identified the ROP-GAP rheostat, which is a key module for adaptation to low oxygen in *Arabidopsis thaliana* through Respiratory Burst NADPH Oxidase Homologs (RBOH)-mediation and ROP GTPase-dependent regulation of reactive oxygen species homeostasis as an early events in scald development^2^. An alternative scenario showed that the pathway of chlorogenic acid and its subsequent oxidation by polyphenoloxidase is responsible for scald browning symptoms. In line with this hypothesis, an anti-apoptotic regulatory mechanism for the compartmentation of scald symptoms in ‘Granny Smith’ apple was proposed^7^. Enhanced methanol and methyl ester levels alongside the upregulation of pectin methylesterases, are also involved in symptom development^4^. There is also evidence that specific metabolomic changes occurred in ‘Granny Smith’ apple prior to actual scald symptom development^8–10^. Global protein analysis revealed that ethylene biosynthesis, phenylpropanoid metabolism, allergens, and sulfur amino acids containing proteins are potentially involved in scald development^11^. Although these studies suggest that α-farnesene oxidation does not act isolated for scald appearance but in concert with various metabolic pathways, our understanding of how these metabolic reprogramming could contribute to scald resistance remains rudimentary. The recent completion of apple genome assembly^12^ provides an unprecedented opportunity to investigate the uncharacterized pathways involved in scald syndrome.

As an initial step to gain insights on scald resistance mechanism, we used the well-established DPA and 1-MCP treatments as experimental tools to characterize scald responses in the scald-sensitive ‘Granny Smith’ apples. These two chemical treatments display similar functions (control scald symptoms) but distinct mechanisms of action (DPA act as antioxidant while 1-MCP as an ethylene inhibitor)^2,13^ and hence, represent a model to study ethylene–dependent and –independent scald responsive mechanisms. Subsequently, we identified novel molecular pathways involved in scald resistance by using proteomic, metabolomic and targeted transcriptomic approaches. Based on this approach, we propose a unified model for how DPA and/or 1-MCP may induce scald resistance in apple.

## Results

### Superficial scald symptoms development

**Figure 1:**
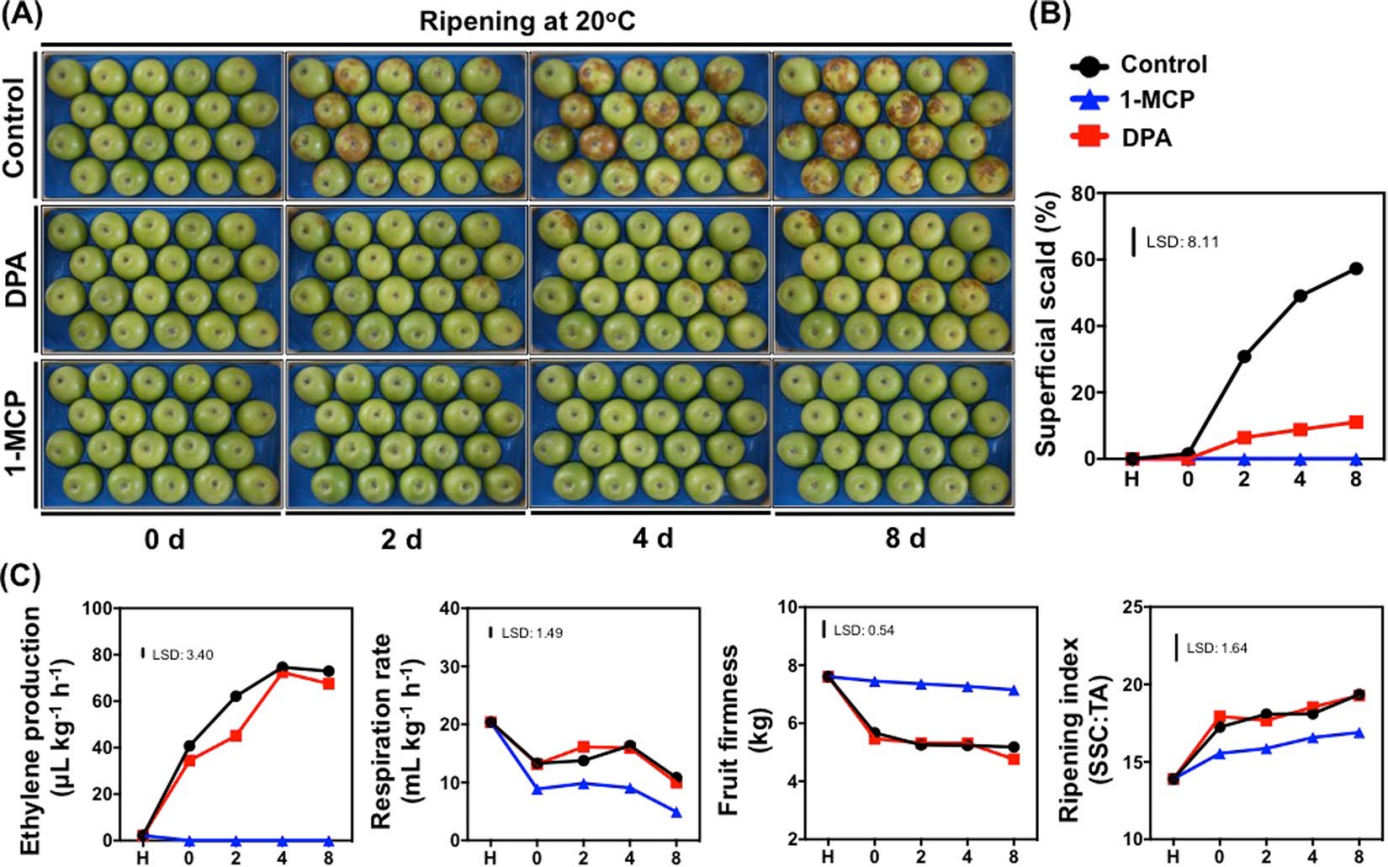
Ripening features of ‘Granny Smith’ apples following DPA and 1-MCP treatments. After harvest, apples were treated with DPA (2000 ppm, 1 min) or 1-MCP (1000 ppm, 24 h, 0 °C), cold stored (0 °C, RH 95%) for 3 months and subsequently transferred at 20 °C to characterize their ripening performance. Phenotypes of apple fruits during various ripening time points at 20 °C showing different expression of scald symptoms development (**A**). Percentage of fruit affected by superficial scald (**B**). Changes of firmness, ripening index (SSC:TA), ethylene production and respiration rate in apple fruit during various ripening time points at 20 °C (**C**). Each value represents the mean of three biological replications of seven fruits analyzed at each ripening stage. The vertical bar represents the least significant difference (LSD, P = 0.05), which was used for means comparison between the different treatments and ripening times.

The DPA/1-MCP-treated and untreated fruits were phenotyped at various ripening stages (0, 2, 4, 8 d at 20 °C) following cold storage (3 months at 0 °C) (Fig. 1A). Several untreated (control) fruits at 2 d ripening and thereafter displayed scald symptoms; up to 57% of untreated apples fruits underwent scald after 8 d ripening. Chemical treatments with either DPA or 1-MCP substantially reduced such visible injury. At 8 d ripening, scald percentage was reduced to 11.4% in DPA-treated apples while no visual scald symptoms (0%) were observed in fruit exposed to 1-MCP (Fig. 1B). At this time point, DPA-treated fruits increased their surface lightness (L*), while 1-MCP-treated fruits decreased both color b (b*) and Chroma in comparison to control; in addition, hue angle was increased in response to 1-MCP treatment (Supplementary Fig. S1).

### Effect of DPA and 1-MCP on Granny Smith apple ripening physiology

To examine the ripening physiology of ‘Granny Smith’ apples in response to DPA and 1-MCP treatments fruits were sampled at harvest and across ripening times. The apple ripening process was significantly delayed by 1-MCP, as documented by the decreased fruit firmness compared with control as well as the lower ripening index (due to lower acidity, data not shown) after 8 d ripening at 20 °C (Fig. 1C). In response to 1-MCP, no ethylene production was detected during ripening period, while ethylene level was not affected by DPA treatment, except at 2 d that was significantly lower in relation to control. Moreover, no significant difference in firmness level was observed in DPA-treated fruits during the whole ripening period (Fig. 1C). Although respiration rate was unaffected by DPA when compared with control, in contrast a lower respiration rate was observed in 1-MCP treated fruits (Fig. 1C).

### DPA and 1-MCP affect protein accumulation levels in different ways

**Figure 2:**
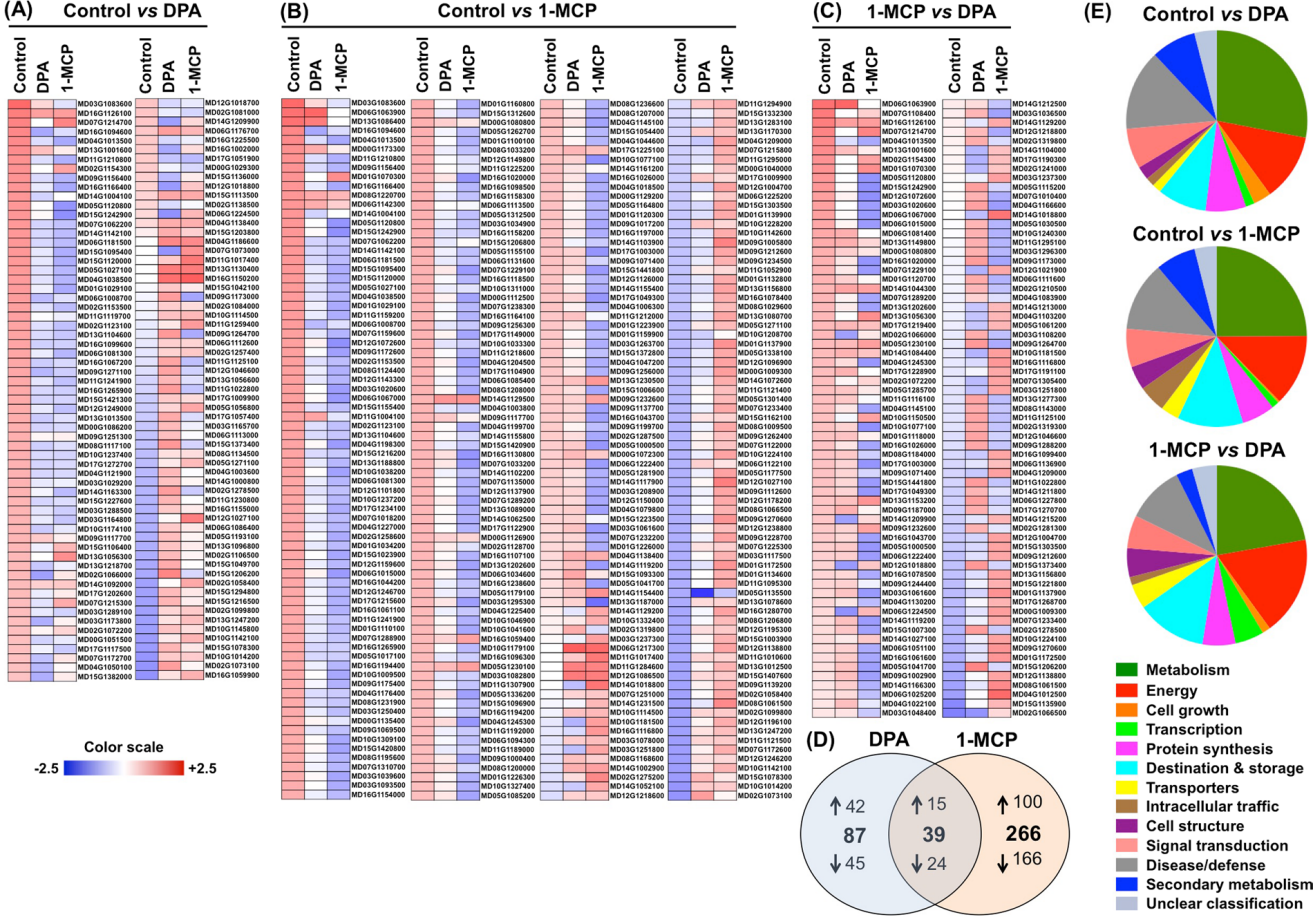
Apple skin protein alternations due to DPA and 1-MCP treatments. The heat map depicts the relative level/abundance of each protein at cold storage for 3 months and subsequently transferred at 20 °C in order to characterize their ripening. (**A**) depicts the relative level/abundance of proteins Z-score fold change that significantly altered uniquely by DPA treatment compared to control, (**B**) depicts the relative level/abundance of proteins Z-score fold change that significantly altered uniquely by 1-MCP treatment compared to control, (**C**) depicts the relative level/abundance of proteins Z-score fold change that significantly altered by 1-MCP compared to DPA treatment, (**D**) Venn diagram which presents the total number of proteins that up- or down-accumulated by DPA and 1-MCP treatments compared to control and (**Ε**) functional classification of the identified proteins according to^73^. Z-score fold change values are shown on a color scale that is proportional to the abundance of each identified protein. Mean values of four independent determinations for each treatment were analyzed between the treatment and the control using the Perseus software. Details of each protein are summarized in Supplementary Table 1.

Considering the phenotypic data (Fig. 1A,B) in combination with the climacteric rise in ethylene production observed in both untreated-control and DPA-treated fruit (Fig. 1C), a scald-proteome analysis was performed following 4-d exposure to room temperature. The proteomic data disclosed 2905 confidently identified and Label-Free Quantification (LFQ) quantified proteins of the apple skin tissue. The three different proteomes were processed statistically using the Perseus software (version 1.6.0.2) to reveal the up- and/or down-regulated proteins as a result of the chemical treatments. A Welch’s T-test with p-value used for truncation (less than 0.05) was performed, comparing DPA treatment vs control, 1-MCP treatment vs control as well as DPA treatment vs 1-MCP treatment. As a result, the accumulation levels of a total number of 392 proteins were altered among control, DPA and 1-MCP (Fig. 2A–D). Particularly, we found that the abundance of 87 proteins was specifically induced or repressed by DPA (42 were up-accumulated and 45 were down-accumulated) (Fig. 2A,D) while 266 proteins were changed (100 were up-accumulated and 166 were down-accumulated) by 1-MCP (Fig. 2B,D) and therefore could be involved in scald resistance. Notably, levels of 39 apple proteins were commonly affected (15 were up-accumulated and 24 were down-accumulated) by both DPA and 1-MCP (Fig. 2C,D). A close examination of the identified DPA and 1-MCP-affected proteins **(**Fig. 2A, Supplementary Table S1, Supplementary Table S6) indicated that several of them are involved in the sulfur metabolism, including cysteine, methionine, folates, methionine cycle and transmethylation reactions, and glutathione. This is the case, for example, for glutathione dehydrogenase/transferase, lactoylglutathione lyase, caffeoyl-CoA *O*-methyltransferase, cystathionine beta-synthase, *S*-adenosyl-L-homocysteine hydrolase, *S*-adenosyl-L-methionine-dependent methyltransferase, glutathione peroxidase (3 identifications), betaine-aldehyde dehydrogenase, sterol 24-C-methyltransferase, L-3-cyanoalanine synthase/cysteine synthase, adenosine kinase, glycine dehydrogenase, glycine hydroxymethyltransferase, cystathionine gamma-synthase, glutathione dehydrogenase/transferase, *S*-(hydroxymethyl)glutathione dehydrogenase, glutamate–cysteine ligase, protein-L-isoaspartate (D-aspartate) *O*-methyltransferase, 24-methylenesterol C-methyltransferase, aspartate aminotransferase, sarcosine oxidase, protein arginine methyltransferase, cysteine desulfurase, and cysteine synthase A **(**Fig. 2A, Supplementary Table S1).

An increase in abundance of various proteins, such as dihydroxy-acid dehydratase, D-aminoacyl-tRNA deacylase and phospholipase C was exclusively observed in fruit exposed to DPA **(**Fig. 2A, Supplementary Table S1). Meanwhile, accumulation levels of several proteins, including regulator of chromosome condensation (RCC1) protein, plasminogen activator inhibitor 1 RNA-binding protein (PAI*-*1) and HSP20 were specifically decreased by DPA. Furthermore, the accumulation levels of a group of apple proteins, such as fructose-bisphosphate aldolase, triosephosphate isomerase and mannose-1-phosphate guanylyltransferase were solely increased by 1-MCP. Finally, the accumulation of several proteins, including serine/threonine-protein phosphatase PP1 catalytic subunit, pathogenesis-related 4 and GDSL lipase 1 was depressed by 1-MCP. In addition to the differentially accumulated proteins, a common proteomic function in DPA and 1-MCP appplication was noticed, which includes, among others, several proteins involved in antioxidant (i.e. L-ascorbate peroxidase and monodehydroascorbate reductase) and ubiquitin*-*proteasome systems (i.e. ubiquitin carboxyl-terminal hydrolase L3 and 26 S proteasome regulatory subunit N12) **(**Fig. 2A, Supplementary Table S1).

### Scald-associated gene expression analysis

**Figure 3:**
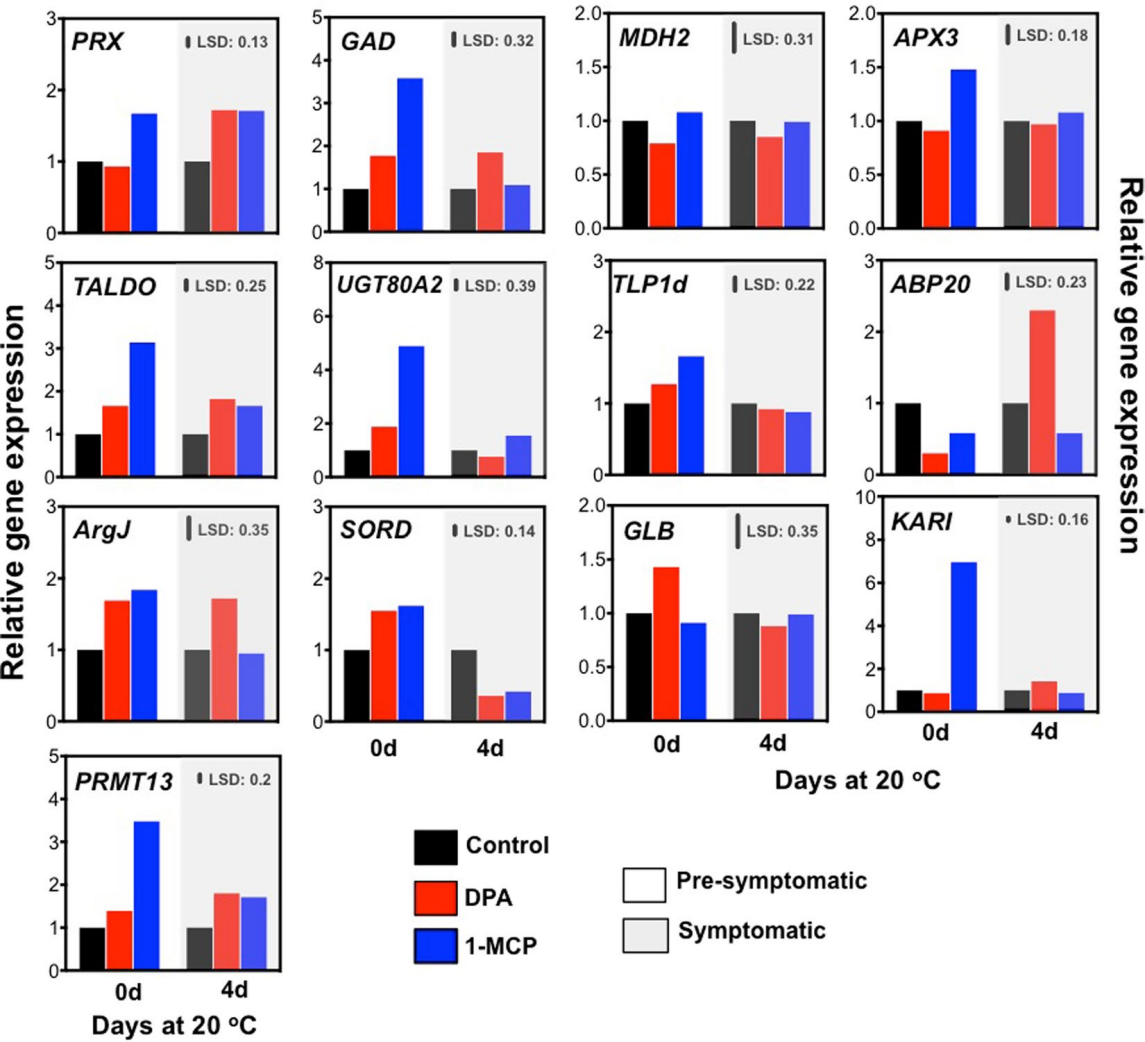
Gene expression analysis in pre-symptomatic and symptomatic apple skin tissue. qRT-PCR analysis was performed during ripening on tissue sampled just after 3-months of cold storage (0 d at 20 °C; pre-symptomatic period), and after 4 d ripening at 20 °C (symptomatic period). Additional experimental details as described in Fig. 1. Pooled samples from three independent skin tissues were analyzed for each (three) biological replicates. Gene name abbreviations: *PRX*: peroxidase, *GAD*: glutamate decarboxylase, *MDH2*: malate dehydrogenase, *APX3*: L-ascorbate peroxidase, *TALDO*: transaldolase, *UGT80A2*: sterol 3-beta-glucosyltransferase, *TLP1d*: thaumatin, *ABP20*: auxin-binding protein ABP20, *ArgJ*: arginine biosynthesis bifunctional protein ArgJ, *SORD*: sorbitol dehydrogenase, *GLB*: β-galactosidase, *KARI*: ketol-acid reductoisomerase, *PRMT13*: histone-arginine methyltransferase 1.3.

To further characterize candidate mechanisms involved in scald, the expression of genes encoding proteins that affected by DPA and/or 1-MCP treatments [e.g. peroxidase (*PRX*), malate dehydrogenase (*MDH2*), L-ascorbate peroxidase 3 (*APX3*), transaldolase (*TALDO*) and *β*-galactosidase (*GLB*) was monitored by quantitative RT-PCR at 0- and 4-d ripening (Fig. 3). In addition, we also investigated at these time points the expression level of a group of genes known to be involved in various cellular process potentially linked to scald, namely sterol 3-beta-glucosyltransferase (*UGT80A2*)^14^, thaumatin-like protein1b (*TLP1b*)^15^, glutamate decarboxylase (*GAD*)^16^, auxin-binding protein (*ABP20*)^17^, arginine biosynthesis bifunctional protein (*ArgJ*)^18^, sorbitol dehydrogenase (*SORD*)^19^, galactosidase beta (*GLB*)^20^, ketol*-*acid reductoisomerase (*KARI*)^21^ and histone-arginine methyltransferase 1.3 (*PRMT13*)^22^ (Fig. 3). The first sampling coincided with protein analysis (Fig. 2) and corresponded before scald visual appearance (0 d at 20 °C, Fig. 1A,B; herein defined as ‘pro-symptomatic period’). Sampling on 4 d at 20 °C corresponded to the time point at which all scald-affected fruits displayed scald symptoms (Fig. 1A,B) herein defined as ‘symptomatic period’. Comparison between proteomic and targeted transcriptomic datasets revealed that most of the proteins and their corresponding transcripts, such as *GAD* and *UGT80A2* showed a similar pattern before scald appearance (Figs 2A,C and 3). This protein and transcript profile comparison also depicted some divergent patterns, as observed for *TALDO* and *APX3* indicative of possible post-transcriptional events (Figs 2A,C and 3). Gene expression data further showed that the majority of the selected transcripts were specifically induced by DPA and 1-MCP treatments at 0 d of ripening. For example, expression of *ABP20*, *ArgJ* and *GLB* were up-regulated by DPA at 0-d while that of *KARI* and *PRMT13* was stimulated by 1-MCP (Fig. 3).

### Cytosine methylation-based epigenetic regulation in apple skin tissue

**Figure 4:**
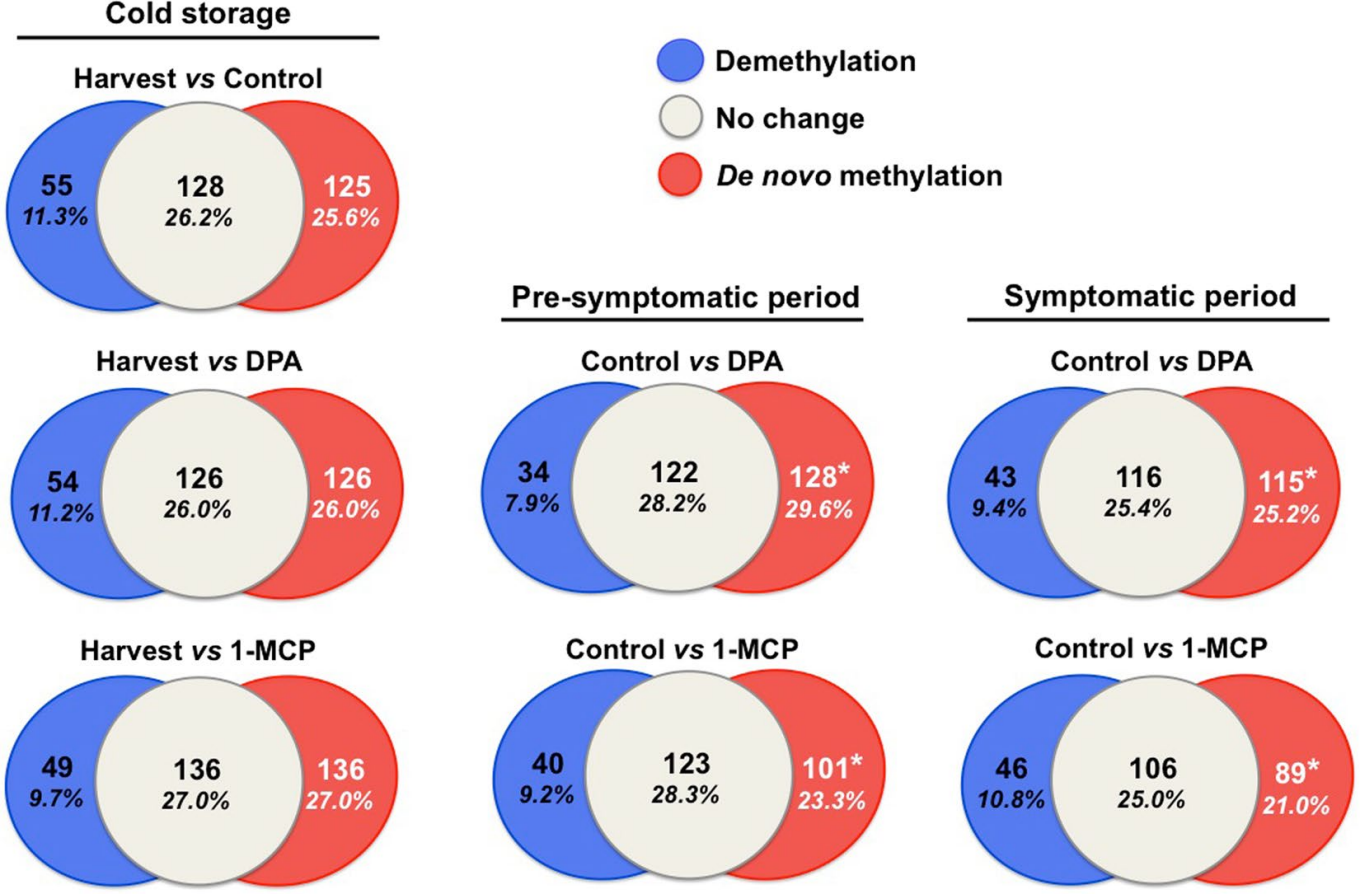
Venn diagrams for global methylation based on relative levels at randomly sampled at 5′- CCGG -3′ sites with the MSAP marker in apple skin tissue (Supplementary Table 5). Methylation was analyzed at harvest, prior to scald visualization (pre-symptomatic period; 0 d at 20 °C), and during scald development (symptomatic period; 4 d at 20 °C). Additional experimental details as described in Fig. 1. The percentages of each methylation status (de novo methylation, demethylation and unchanged) from harvest, control and DPA/1-MCP treatments are also shown. Pooled samples from three independent skin tissues were analyzed for each (three) biological replicates. Data represent mean values from three technical replications for each biological sample. Values that significantly different (P < 0.05) from the harvest/control are marked with an asterisk (*).

To study DNA methylation changes (i.e., *de novo* methylation and demethylation) across apple scald phenotypes (Fig. 1A), the Methylation-Sensitive amplification polymorphism (MSAP) analysis was employed to analyze cytosine methylation at harvest as well as at pre-symptomatic and symptomatic periods (Fig. 4). The MSAP method detects the DNA methylation status of 5′-CCGG-3′sites employing two pairs of isoschizomers *EcoRI/HpaII* and *EcoRI/MspI* that have different sensitivity to methylation at the inner or outer cytosines. The DNA methylation pattern at 5′-CCGG-3′ sites is determined by scoring the presence or absence of bands after *EcoRI/HpaII* and *EcoRI/MspI* digestion of genomic DNA and subsequent PCR amplification. Differences in PCR products obtained from the different treatments reflect different methylation states at the restriction site of cytosines^23^. Following cold exposure, no *de novo* methylation or demethylation level alteration was detected in apples exposed to chemical treatments compared to harvest (Fig. 4). Remarkably, DNA methylation differences between control and chemical-treated fruits at two post-cold stages were detected. At pre-symptomatic period, methylation level in skin exposed to DPA or 1-MCP was increased by 29.6% (128 methylated regions) and 23.3% (101 methylated regions) compared to control, respectively. Similarly, evaluated at symptomatic period, DPA or 1-MCP treatment increased DNA methylation (methylation levels 25.2% with 115 differentially methylated loci; methylation levels 21.0% with 89 differentially methylated loci, respectively) compared with control. The overall methylation level in DPA was consistently higher than that in 1-MCP under both post-cold conditions (Fig. 4).

### Metabolomic transitions during scald development

**Figure 5:**
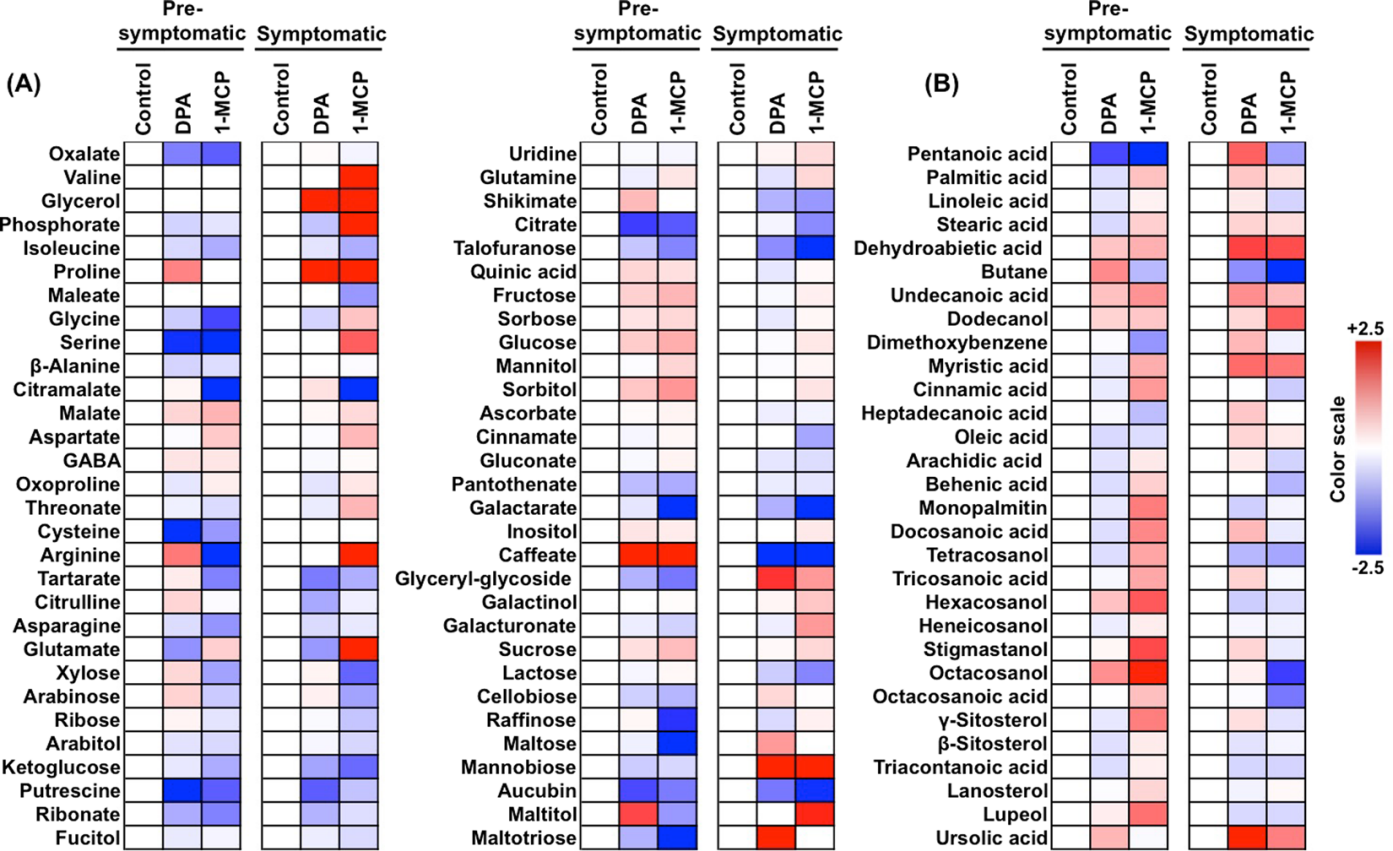
Polar (**A**) and non-polar (**B**) metabolic changes pictured as heat map profiles in apple skin exposed to DPA and 1-MCP treatments. The heat maps depicted the relative level of each metabolite compared to the control after 3 months of cold storage (pre-symptomatic; 0 d at 20 °C) and subsequently after transferred at 20 °C to characterize their ripening (symptomatic; 4 d at 20 °C). Fold change values are shown on a color scale that is proportional to the ratio of each identified metabolite. Mean values of three independent measurements for each treatment were analyzed between the treatment and the control. Relative values for each metabolite mean are provided in Supplementary Table 3.

**Figure 6:**
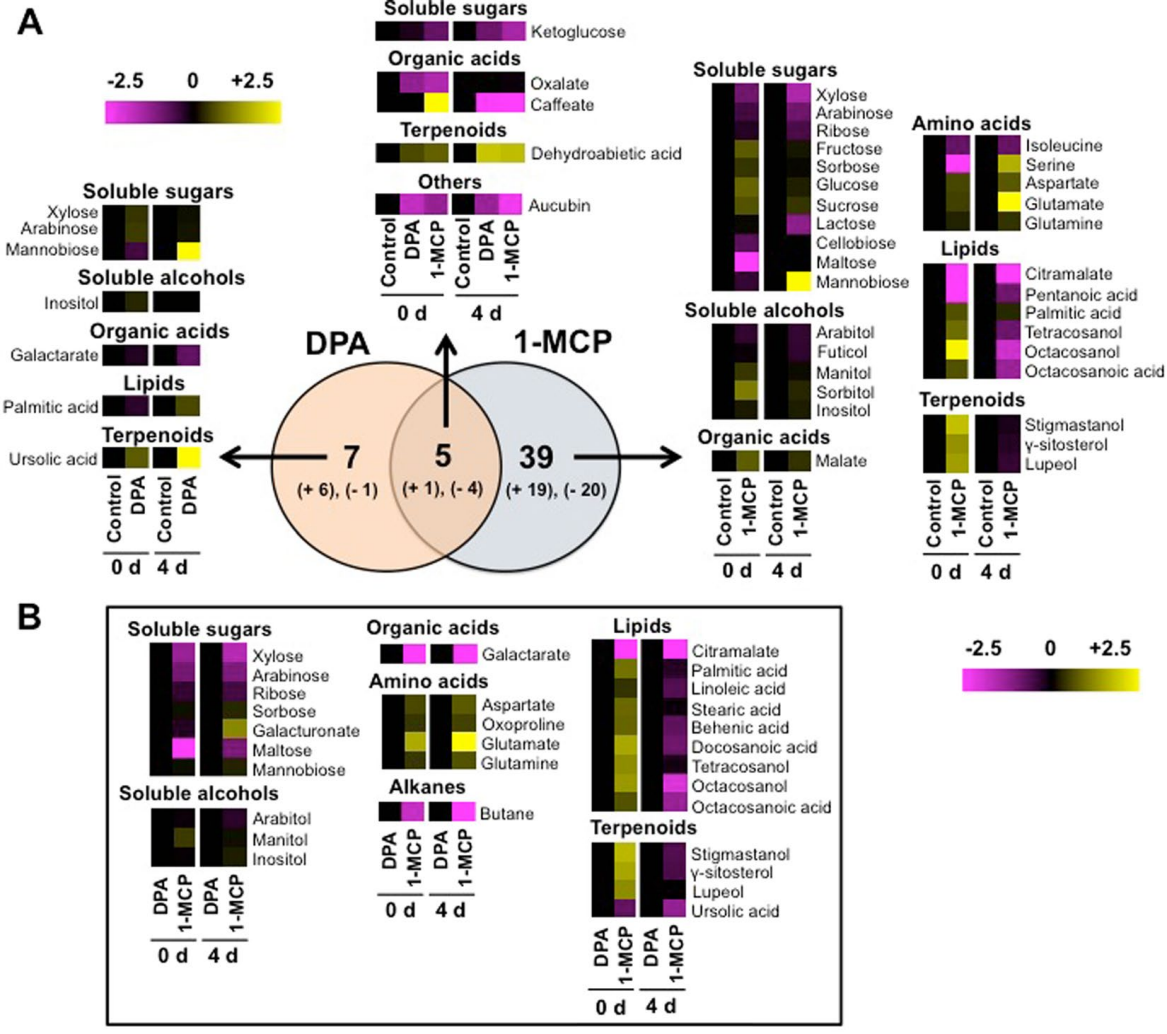
Metabolic changes pictured as heat map profiles in apple skin exposed to DPA and 1-MCP treatments. The graph depicts (**A**) the relative level of each metabolite after DPA or 1-MCP treatment compared to the control. (**B**) Fold change values are shown on a color scale, which is proportional to the mean of each identified metabolite. Mean values of three independent determinations for each treatment were expressed as ratios between the treatment and control using the Multi-Experiment Viewer software (version 4.4.1). Relative values for each metabolite mean are provided in Supplementary Table 3. Metabolites were grouped according to their classification as given in Supplementary Table 4.

To link skin metabolism to scald development, contents of polar (Fig. 5A) and non-polar (Fig. 5B) metabolites were measured at pro-symptomatic (0-d) and symptomatic periods (4-d). Using a GC–MS analysis, 90 compounds were quantified that correspond to lipids (24%), soluble sugars (18%), organic acids (17%), amino acids (12%), terpenoids (8%) and sugar alcohols (7%). From the total number of identified metabolites, the contents of 46 polar and non-polar metabolites were altered between DPA or 1-MCP and control (Fig. 6A). Particularly, the comparison between chemical treatments and control revealed the existence of three groups of metabolites. The first group consisted of 7 metabolites, which were only DPA-responsive and for which level was unaffected by 1-MCP (Fig. 6A). Among these exclusively DPA-affected metabolites, the level of only one (galactarate) was decreased while that for 6 metabolites were increased in comparison to control fruit. DPA specific metabolites include soluble sugars (xylose, arabinose, mannobiose), soluble alcohols (inositol), organic acids (galactarate), lipids (palmitic acid) and terpenoids (ursolic acid). The second group was composed of 39 metabolites the accumulation level of which was changed only by 1-MCP (Fig. 6A). Of these 1-MCP exclusively affected metabolites, 19 showed increased levels while 20 showed decreased levels. These 1-MCP specific metabolites included, among others, several soluble sugars (eg. lactose, ribose), soluble alcohols (eg. sorbitol, arabitol), lipids (pentanoic acid, octacosanoic acid), amino acids (eg. isoleucine, serine) and terpenoids (eg. stigmastanol, *γ*-sitosterol). The third group included 5 metabolites whose levels were affected by both DPA and 1-MCP, and contained one compound (dehydroabietic acid) whose level was increased and 4 metabolites whose level were decreased (Fig. 6A). A further comparison between DPA and 1-MCP treated fruits indicated that the contents of a total of 29 metabolites were varied (Fig. 6B). The largest group of these differentially accumulated metabolites was related to lipids (eg. citramalate, docosanoic acid). The metabolism cluster of sugar metabolites (eg. galacturonate, maltose) was the second most abundant category followed by terpenoids (eg. stigmastanol, ursolic acid) (Fig. 6B).

**Figure 7:**
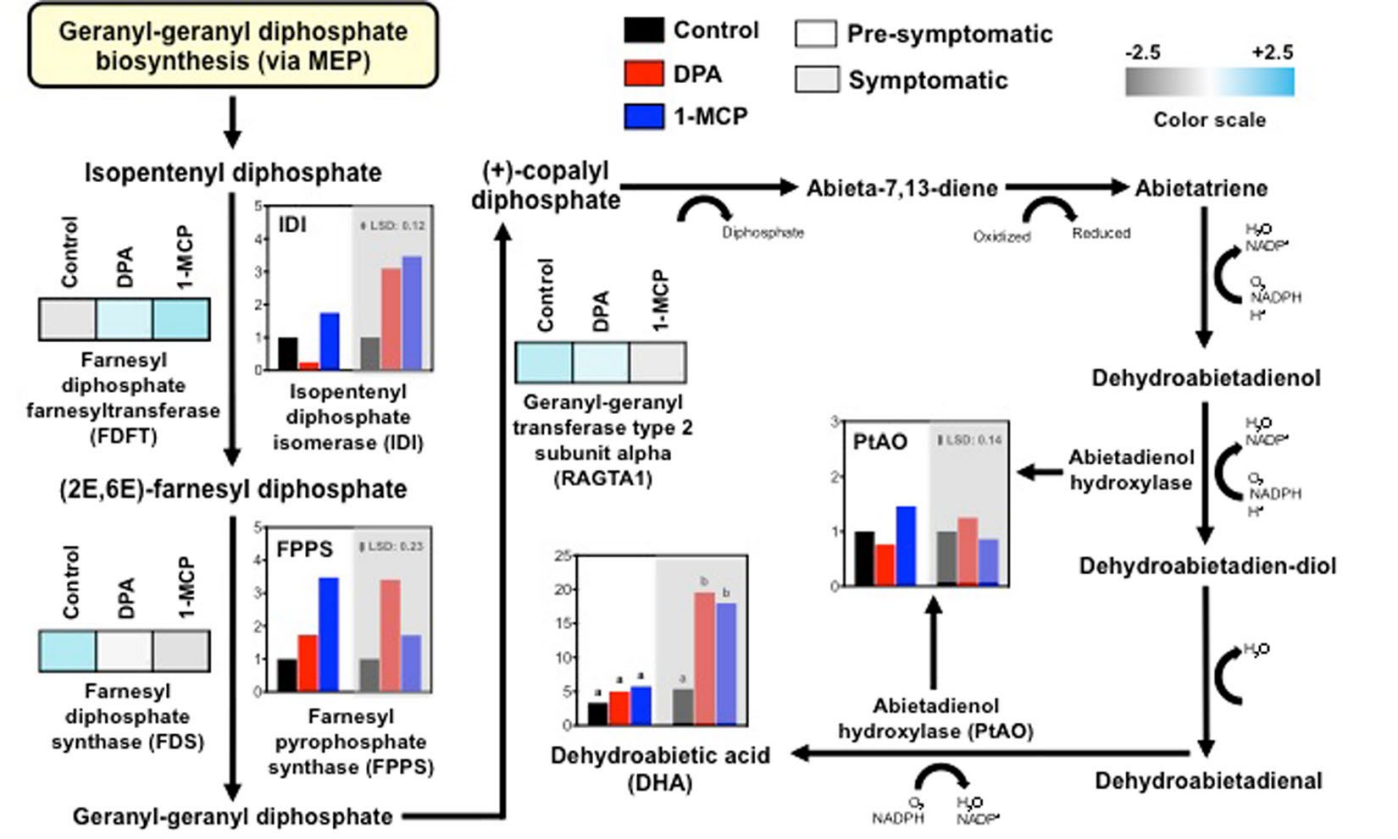
Schematic representation of the plant biosynthetic pathway of geranyl-geranyl diphosphate biosynthesis via mevalonate pathway. Metabolic differences and transcriptional regulation were presented as vertical bars that represent the relative abundance/expression of primary metabolites/genes analyzed prior to DPA and 1-MCP treatments and after 3-months cold storage (pre-symptomatic; 0 d at 20 °C) and 4 d ripening at 20 °C (symptomatic). Additional experimental details as described in Fig. 1. The vertical bar in each particular gene figure plate represents the least significant difference (LSD, P = 0.05), which was used for means comparison between the different treatments and ripening times. Different letters in each metabolite figure indicate significant differences among the treatments according to Duncan’s multiple range test and Student’s t-test.

To gain a comparative insight into the similarities and differences into how skin tissue built scald resistance, the metabolites of apple fruits treated with DPA or 1-MCP were compared in more detail. Given that dehydroabietic acid (DHA) was strongly induced by both DPA and 1-MCP at symptomatic period (Fig. 6, Supplementary Table S4), we generated a metabolic map combined with line graphs of individual gene expression profiles involved in DHA biosynthesis *via* geranyl-geranyl diphosphate reaction (*via* MEP) (Fig. 7). In conjunction with metabolomics analysis, the expression of genes encoding abietadienol hydroxylase (CYP720B1, PtAO), isopentenyl diphosphate isomerase (IDI) and especially farnesyl pyrophosphate synthase (FPPS) was increased by both treatments (Fig. 7). Also, the protein accumulation levels of farnesyl diphosphate farnesyltransferase (FDFT), farnesyl diphosphate synthase (FDS) and geranyl-geranyl transferase type 2 subunit alpha (RAGTA1) protein accumulation level were significantly affected by 1-MCP (Fig. 7, Supplementary Table S1), indicating that the metabolic route of geranyl-geranyl diphosphate biosynthesis represents a common link of scald resistance in response to the two chemical treatments.

**Figure 8:**
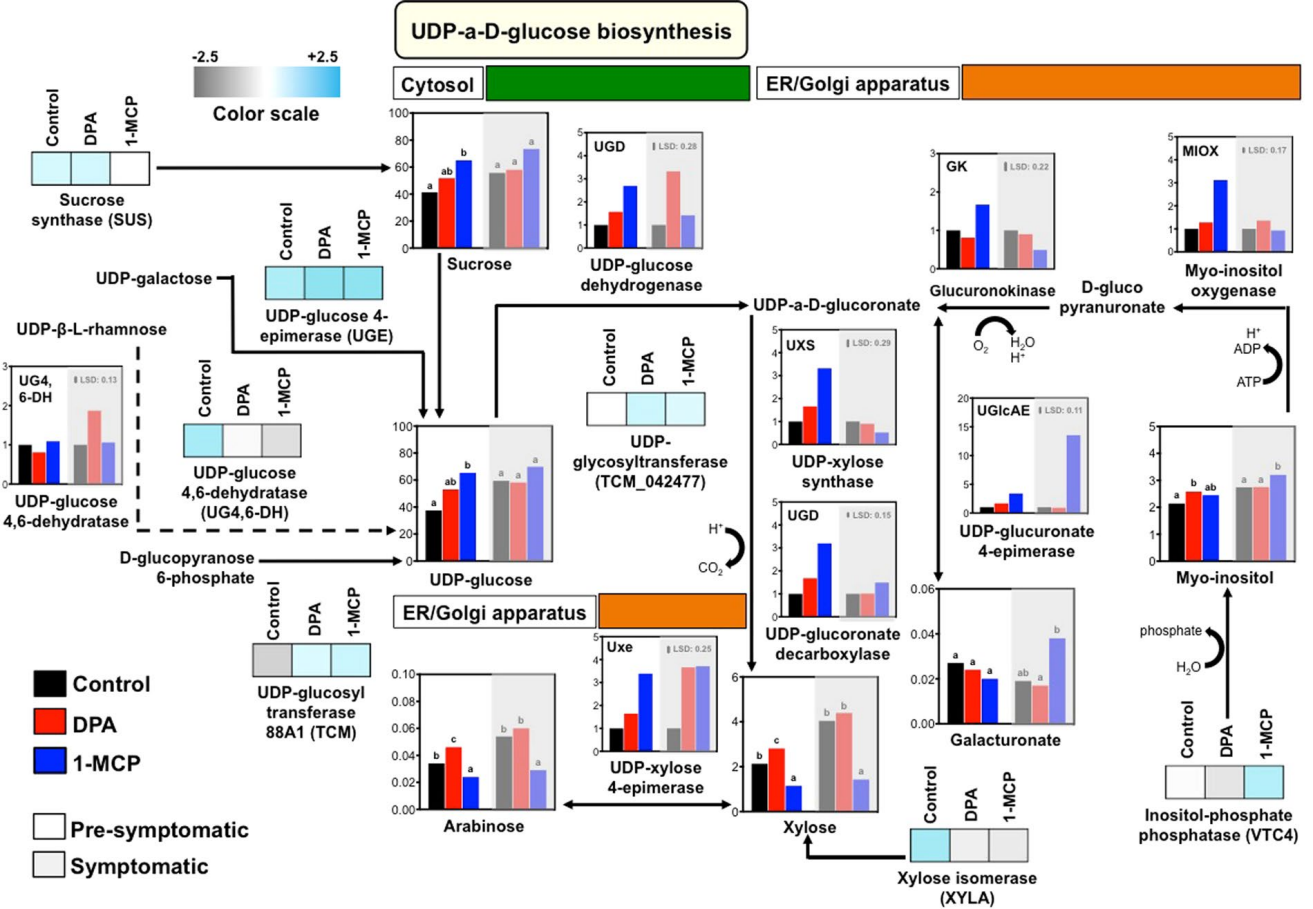
Schematic representation of the plant biosynthetic pathway of UDP-xylose and UDP-arabinose via UDP-a-D-glucose and myo-inositol. Metabolic differences and transcriptional regulation are presented as vertical bars that represent the relative abundance/expression of primary metabolites/genes analyzed prior to DPA and 1-MCP treatments and after 3-months cold storage (pre-symptomatic; 0 d at 20 °C) and 4 d ripening at 20 °C (symptomatic). Additional experimental details as described in Fig. 1. The vertical bar in each particular gene figure plate represents the least significant difference (LSD, P = 0.05), which was used for means comparison between the different treatments and ripening times. Different letters in each metabolite figure indicate significant differences among the treatments according to Duncan’s multiple range test and Student’s t-test.

This study also uncovered that the metabolic profile and the underlying transcriptional analysis of DPA and 1-MCP treated apple fruits differ significantly in many aspects. For example, the level of uridine diphosphate (UDP)-glucuronic acid, UDP-xylose and UDP-arabinose in fruit exposed to DPA was observed to be different from that of 1-MCP under both pro-symptomatic and symptomatic periods (Fig. 6, Supplementary Table S4), pointing that these treatments may regulate conversion of various UDP-sugars in apple skin. Therefore, we decided to study in deeper detail the expression of genes involved in this sugar biosynthesis from intrinsic UDP-glucose by qRT-PCR. Schematic representation of the plant biosynthetic pathway for the production of UDP-sugars is presented in Fig. 8. Data indicated that the expression of the majority of tested genes involved in UDP-D-glucose biosynthesis, including *UDP-glucuronate 4-epimerase* (*UGlcAE*), *UDP-xylose 4-epimerase* (*Uxe*), *UDP-glucoronate decarboxylase* (*UGD*) and *UDP-xylose synthase* (*UXS*) was up-regulated by DPA. Meanwhile, transcript levels of *UDP-xylose 4-epimerase* (*Uxe*), *glucoronokinase* (*GK*), *myo-inositol oxygenase* (*MIOX*), *UDP-xylose 4-epimerase* (*Uxe*), *UDP-glucoronate decarboxylase* (*UGD*) and *UDP-xylose synthase* (*UXS*) were strongly induced by 1-MCP at pre-symptomatic period (Fig. 8), suggesting that activation of UDP-D-glucose metabolism may be an important episode linked with scald resistance. In support of this assumption, skin tissue exposed to 1-MCP altered sucrose synthase (SUS), UDP-glucosyl transferase 88A1 (TCM), UDP-glycosyltransferase (TCM_042477), inositol-phosphate phosphatase (VTC4), xylose isomerase (XYLA) protein level at pre-symptomatic period (Fig. 2A, Supplementary Table S1).

## Discussion

This study characterized cellular changes occurring during superficial scald resistance in ‘Granny Smith’ apple fruits following DPA and 1-MCP treatments, a model system to investigate scald. Given that ethylene has long been implicated in scald development^10,24^, along with our observation that both DPA and 1-MCP treatments were effective to prevent scald symptoms (Fig. 1A,B), although displaying differential ripening behavior, including ethylene emission (Fig. 1C), the system presently used provide us the opportunity to discriminate between ethylene–dependent and –independent scald etiology in apples. The underlying rationale was that this kind of studies, comprising multi-omics approaches, could provide new insights into processes underlying scald development, and a comprehensive view of the major pathways required for scald resistance.

Proteomic analysis indicated that a reprogramming of apple metabolism occurs after cold exposure and during the onset of scald development (Fig. 2, Supplementary Table S1), possibly leading to phenotypic scald-defects (Fig. 1A). The abundance of several of these potentially scald-affected proteins has also been shown to vary in several fruit systems subjected to post-cold conditions (e.g. tubulin alpha, chalcone isomerase, prolyl-tRNA synthetase, carbamoyl-phosphate synthase large subunit, alanine transaminase, malonyl-CoA/methylmalonyl-CoA synthetase, ubiquitin carboxyl-terminal hydrolase L3, pyrroline-5-carboxylate reductase, sucrose-6-phosphatase etc.) and may therefore globally play important roles in scald signaling. On the other hand, the present work also characterized several proteins (e.g. BSD domain-containing protein, sarcosine oxidase, syntaxin 1B/2/3, xyloglucan/xyloglucosyl transferase, asparaginyl-tRNA synthetase, rubredoxin family protein, melibiase family protein, phenylpyruvate tautomerase, glycerophosphoryl diester phosphodiesterase, urease accessory protein G etc.) that were not previously found to be scald-related in various proteomic studies^11,25,26^. Thus, the present data (Supplementary Table S1) combined with previous results may serve as a framework for scald-associated protein responses. We also demonstrate that protein repression is essential for scald ‘preventing strategy’ since an extensive down-accumulation of several proteins was observed following DPA and 1-MCP treatments (Fig. 2). For example, the abundance of several proteins related to defense, such as betaine-aldehyde dehydrogenase, Cu/Zn-superoxide dismutase and catalase, was decreased by chemical treatments (Supplementary Table S1; Fig. 2); such behavior might represent a scald-resistance strategy to establish a new homeostasis in skin metabolism after a prolonged cold period. Alternatively, these proteins were downregulated because DPA and 1-MCP treatments reduced cold stress severity and thus several signaling molecules prevented the accumulation of defense proteins.

Current data further uncovered that several proteins were differentially accumulated by DPA and 1-MCP. It is therefore conceivable that some of these proteins could be differentially regulated by chemical treatments due to their distinct effect on ripening (Fig. 1C). Such a distinct ripening behavior could explain the observed accumulation of *β*-galactosidase, an enzyme whose role against cell wall degradation is well documented^27,28^, following 1-MCP application (Fig. 2, Supplementary Table S1). Another interesting example is the decrease in *γ*-carbonic anhydrase (CA) that could, at least partially, explain the apple fruit respiration pattern (Fig. 1C), since this mitochondrial enzyme catalyzes the reversible hydration of CO_2_ to HCO_3_^−^^29^. Nevertheless, a second explanation also arises regarding the physiological significance of these 1-MCP-responsive proteins towards cold exposure and subsequent scald appearance. For instance, we found that transcript and protein levels of transaldolase (*TALDO*) as well as the abundance of cold-regulated 47 (COR47) (Fig. 2, Supplementary Table S1) were induced by 1-MCP. Although the exact role of TALDO and COR47 in scald biology remains uncertain, there is evidence indicating that TALDO participates in the production of secondary-defense metabolites against abiotic stress^30^ while COR47 is directly involved in plant cold responses^31^. In addition, the fact that several Ras-related proteins (Rab family), such as Rab-11A/Rab-18/Rab-5C/Rab-7A were strongly depressed by 1-MCP (Fig. 2, Supplementary Table S1), raises new possibilities about the role of Rab proteins in scald because these proteins (1) are regulated by ethylene^32^, (2) serve as signaling nodes in response to diverse external environment stimuli^33^, and (3) act as key regulators of intracellular vesicular transport and the trafficking of proteins between different organelles^34^. In *Arabidopsis*, more than 17% of all gene products are predicted to enter the endomembrane system^35^ and each must be transported to its correct destination. Hence, Rab family proteins could regulate cytoplasmic signaling networks that control gene expression and regulation of scald hallmarks. In support to the role of membrane trafficking and vesicular transport in scald resistance, our data disclosed that the abundance of intracellular Ras group-related LRR 9, trafficking protein particle complex subunit 3 and vesicle transport protein SEC. 22 was strongly affected by 1-MCP (Fig. 2, Supplementary Table S1).

Our results also demonstrated that targets of DPA are involved in various pathways for biosynthesis, metabolism, and signaling, such as translation, redox homeostasis and protein synthesis, signifying the influence of DPA to a wider range of apple metabolism. In particular, DPA appears to regulate several translation initiation factors (eIFs), including eukaryotic translation initiation factor 4B1, translation initiation factor 3 subunit F / subunit L and subunit I (Fig. 2, Supplementary Table S1), providing a role for DPA in eIFs-associated scald processes, such as global protein synthesis and preferential production of key proteins involved in adaptation to environmental stress^36^. Meanwhile, the increased level of serine/threonine-protein phosphatase 2B regulatory subunit, along with the transcript *ABP20* up-regulation in DPA-treated apples (Supplementary Table S1; Figs 2 and 3), indicated active adaptation to cold stress since both factors participate in signal transduction under abiotic stresses^37,38^.

Of particular interest is the fact that the abundance of many proteins involved in sulfur metabolism was substantially affected b**y** DPA and 1-MCP applications **(**Fig. 2A, Supplementary Table S1), including cysteine, methionine, folate, and glutathione metabolisms, methionine cycle and transmethylation reactions. It is well known that the biosynthesis of Met in plants is closely coordinated with that for other essential amino acids (e.g. lysine, branched-chain amino acids)^39–41^. It is therefore interesting to note the identification of dihydroxy-acid dehydratase, 2-isopropylmalate synthase, threonine synthase (which is allosterically regulated by AdoMet^42^), L-diaminopimelate aminotransferase, threonine dehydratase, and threonine aldolase (Supplementary Table S1). As it is known that AdoMet serves as a precursor for the synthesis of biotin^39^ it is also interesting to observe that the present study identified the biotin carboxylase subunit of acetyl-CoA carboxylase (ACCase) (2 identifications; Supplemental Table S1), which is the biotin-containing subunit of ACCase. This is of interest as ACCase is the rate-limiting enzyme for lipid synthesis^43^. In this context, it is also worth noting the identification of carbonic anhydrase (Supplemental Table S1), which provides the bicarbonate substrate of ACCase, by catalyzing the hydration of CO2 to bicarbonate. Finally, it is interesting to note the identification of protein-L-isoaspartate (D-aspartate) *O*-methyltransferase (Supplemental Table S1). This enzyme is involved in protein repair during aging^44^, hence contributing to longevity and survival in all organisms. The current identification of this enzyme would suggest its important role in superficial scald disorder, and more generally in the mechanisms regulating shelf life in fruits. Altogether the current study tends to highlight the role of the sulfur metabolism, notably the methionine cycle, as a central mechanism to account for superficial scald disorder.

Recent evidence indicates that a strict genetic and epigenetic control influences the fruit development process^12,45^. Data reported here show that both chemical treatment induced methylation at pre-symptomatic (0 d at 20 °C) and symptomatic period (4 d at 20 °C) (Fig. 5), pointing that DPA and 1-MCP may directly reprogram gene expression by activating DNA methylation, possibly altering the physiognomy of scald signaling. It has recently been shown that differential methylation state of the *ACS1* promoter was linked to internal ethylene concentrations^46^ and CO_2_ injury, a physiological storage disorder that occurs in apple fruit during controlled atmosphere storage. Also, it was demonstrated that DNA methylation changes associated to oxidative stress have a role in melanocyte phenotype^47^. It has been recently proposed that fluctuation of sulfur metabolism and particularly methionine could influence DNA methylation and consequently contribute to the modifications of gene expression^48^. These findings challenge the notion that the scald syndrome is a programmable disorder, which is characterized by epigenetic modifications of key genes when exposed to oxidative stress that usually occur during cold storage and ethylene-driven post-harvest ripening^2,49^. The gene-specific epigenetic influence of DPA and 1-MCP in controlling scald is currently under study in our laboratory.

Comprehensive metabolomic profiling of the pre-to-symptomatic transition identified several metabolites that are commonly affected by DPA and 1-MCP, signifying that these scald-preventing treatments share common scald resistance codes. For instance, a set of metabolites, such as ketoglucose oxalate and caffeate, were commonly changed by DPA and 1-MCP (Fig. 6A), showing that these compounds play critical roles in skin cells by preventing scald; however, none of them was previously linked to this physiological disturbance and therefore their function in scald remains to be determined. Notably, the abundance of dehydroabietic acid (DHA), an abietane-type diterpene resin acid, was strongly induced by the two chemical treatments at pre-symptomatic and symptomatic period (Fig. 6A). In this pathway, isopentenyl diphosphate isomerase (IDI) and farnesyl pyrophosphate synthase (FPPS), that are critical genes involved in scald symptomatology^50^, together with abietadienol hydroxylase (CYP720B1, PtAO) were induced by both DPA and 1-MCP (Fig. 7), suggesting that DHA homoeostasis might reflect a common DPA and 1-MCP-driven scald resistance strategy. Furthermore, the abundances of several proteins involved in DHA biosynthesis, including farnesyl diphosphate farnesyltransferase (FDFT), farnesyl diphosphate synthase (FDS) and geranyl-geranyl transferase type 2 subunit alpha (RAGTA1) were affected by 1-MCP (Fig. 2A, Supplementary Table S1). It is also interesting to note that these enzymes are key intermediates in aroma flavor volatiles biosynthesis in climacteric fruit through terpenoid pathway^51^. The accumulation levels in these enzymes by 1-MCP in ‘Granny Smith’ apples (Fig. 2A) is consistent with previous reports showing that 1-MCP remarkably affected the expression of numerous genes in tomato fruit, especially on several key genes associated with aroma volatile biosynthesis^52^. The link between geranyl-geranyl diphosphate biosynthesis**/**DHA homeostasis and plant stress responses is by no means unprecedented, given that DHA and related gene expression have been proposed as a defense mechanism against biotic stress^53^. However, the physiological role of DHA in cold stress-originated scald events in fruit remains to be clarified.

Apart the common aspects of DPA and 1-MCP action, this study also revealed a large number of different metabolic targets. In this sense, metabolic responsiveness to 1-MCP indicated that this chemical is implicated in the accumulation of soluble sugars (e.g. fructose, glucose, sorbose, sucrose) and organic acids (e.g. malate) levels, in agreement with previous reports^54^. The observed increase in sorbitol by 1-MCP at pro-symptomatic period (Fig. 5A) was consistent with the expression profile of sorbitol dehydrogenase (*SORD*) in fruits exposed to this treatment (Fig. 3) and provides evidence for a role for sorbitol metabolism in scald biology as a cryoprotectant during cold storage by preventing dehydration-induced damage to membranes and proteins, as previously suggested in ‘Granny Smith’ skin^47^. In addition, it has been reported that lines of *A. thaliana* overexpressing the sorbitol dehydrogenase (MdS6PDH) apple gene showed higher accumulation of sorbitol compared with the wild-type (WT) control plants^47^. When *A. thaliana* plants (35 S:MdS6PDH and WT) were grown at cold temperatures, only the transgenic lines survived, while the WT plants underwent severe dehydration damage. This further suggests that sorbitol is an important mechanism for inducing cold acclimation and scald resistance in apple fruit^47^.

It is also notable that 1-MCP promoted isoleucine degradation (Fig. 6A) while ketol-acid reductoisomerase (*KARI*) expression, an enzyme involved in isoleucine biosynthesis, was also affected by 1-MCP (Fig. 3). Thus, isoleucine biosynthesis could be, at least in part, associated to scald development, a feature that could account for the two following possibilities. The first one assumes that isoleucine could be critical for protein structure and function, notably following cold-stress conditions because of their unsubstituted aliphatic side chains with branched alkyl groups^55^. This hypothesis is supported by recent reports in peach fruit showing that isoleucine accumulation confers chilling injury resistance phenotypes^21^. Alternatively, isoleucine may act as signaling molecules to regulate genes expression for stress-related proteins^56^.

The abundances of several non-polar metabolites, including fatty acyls (palmitic acid, octacosanol, tetracosanol, octacosanoic acid) and triterpenoids (*γ*-sitosterol, lupeol, stigmastanol) were increased by 1-MCP after cold exposure, and thereafter decreased during ripening (Fig. 6A,B). The importance of 1-MCP-dependent sterol glucosides signaling in scald resistance is further supported by the observation that the expression of sterol 3-beta-glucosyltransferase (*UGT80A2*), which catalyzes the synthesis of steryl glycosides (SGs) and which is required for suberin accumulation and cutin formation^14^, was strongly induced by 1-MCP prior to scald appearance (Fig. 3). Therefore, it is likely that this specific 1-MCP regulation could be related to cutin, suberine and wax biosynthesis. While the relationships between changes in wax composition and scald symptoms remain unknown, it is likely that these biochemical modifications affect the accumulation and diffusion of volatiles, such as *α*-farnesene^4^. Previous researches^57^ suggested that the lack of dependence on airborne *α*-farnesene concentrations with those in the wax results in changing the wax composition; such changes could alter the rate of diffusion through the cuticle diffusion of *α*-farnesene from its site of synthesis in the peel to its site of evaporation from the outer surface of the wax — factors that are frequently associated with scald development^4,58^. In this regard, the 1-MCP-accociated decrease of pentanoic acid (Fig. 6A), which participate in fatty acid *α*- and *β*-oxidation as well as in alkanes oxidation^59^, could be closely related to *α*-farnesene oxidation since *α*-farnesene remains in the lipid phase of the cellular environment^4^. This scenario is also supported by the fact that the enzyme *α*-farnesene synthase (MdAFS1) (MD10G1311000) was repressed in 1-MCP treated apples compared to control (Fig. 2A, Supplementary Table S1), a feature that may explain the observed scald phenotypes (Fig. 1A). Nevertheless, several non-polar metabolites, such as palmitic and ursolic acid, which are highly connected to cutin, suberine and wax biosynthesis^60^, disclosed decreased levels in apples exposed to DPA (Fig. 6A,B). This is an interesting observation since *α*-farnesene accumulates mostly in the wax layer and not at the inner hydrophilic layers of the cuticle or the epidermal cell walls, whereas scald symptoms develop in hypodermal and epidermal cells rather than in lipophilic cuticle^61^.

Another interesting finding is the induction of both UDP-xylose and UDP-arabinose upon DPA exposure (Fig. 6A,B). Because UDP-xylose and UDP-arabinose are produced from UDP-glucose^62^, we examined the expression of genes involved in several steps of this biosynthetic pathways; an alternative route for the production of galacturonate *via* myo-inositol was also characterized (Fig. 8). Data indicated that *UDP-glucuronate 4-epimerase* (*UGlcAE*), *UDP-xylose 4-epimerase* (*Uxe*), *UDP-glucoronate decarboxylase* (*UGD*) and *UDP-xylose synthase* (*UXS*) (Fig. 8) along with abundance of several UDP-D-glucose-related protein, such as UDP-glucosyl transferase 88A1 (TCM) UDP and glycosyltransferase (TCM_042477) (Fig. 2, Supplementary Table S1) were induced by DPA. Also, the expression of UDP-xylose 4-epimerase (Uxe), glucoronokinase (GK), myo-inositol oxygenase (MIOX), UDP-glucoronate decarboxylase (UGD) and UDP-xylose synthase (UXS) (Fig. 8) together with the abundance of UDP-glucosyl transferase 88A1 (TCM), UDP-glycosyltransferase (TCM_042477) and inositol-phosphate phosphatase (VTC4) were up-regulated by 1-MCP, particularly prior to scald appearance (Fig. 2, Supplementary Table S1). It is noted that the observed dissimilarities between the effect of DPA and 1-MCP on UDP-xylose and UDP-arabinose biosynthesis may result from differences in the cellular compartment in which UDP-arabinose is generated by the respective pathway (Fig. 8). These results support the existence of a link between UDP-D-glucose regulatory events and alternative myo-inositol pathways leading to scald resistance. Increased myo-inositol level in response to 1-MCP has been reported in ‘Empire’ apple fruit at post-cold ripening following different storage temperatures^45^, further supporting that 1-MCP treatment could modulate subsequent scald responses. The active nucleotide sugar interconversion pathways in scald resistance apple fruits led us to draw two conclusions. First of all, these nucleotide sugars are likely to perform a function as cell wall components in order to keep the cell wall flexibility during post-cold ripening^63^. Second, apple skin following cold exposure could activate a UDP-sugar recycling control system, which might then trigger programmed cell death (PCD), indirectly observed as superficial scald symptoms. Consistent with this hypothesis is the fact that virus-mediated silencing of UDP-xylose synthases in tobacco resulted in cell death and phenotypic disturbance^64^ while an active role for PCD as part of a series of processes leading to scald appearance has been recently proposed^7,65^. In support to putative role of PCD in scald phenomenon, our data showed that a large group of proteins (14 proteins) associated with ubiquitin*-*proteasome system undergo dramatic shifts in their accumulation following chemical treatments (Fig. 2, Supplementary Table S1); however, further research is needed to unravel this scenario. Finally, it is interesting to note that our transcript analysis also showed that the expression profiles of several genes, including arginine biosynthesis bifunctional protein (*ArgJ*), sorbitol dehydrogenase (*SORD*), trifunctional galactosidase beta (*GLB*) and histone-arginine methyltransferase 1.3 (*PRMT13*) were remarkably affected by chemical treatments (Fig. 3), suggesting that scald resistance pathway is likely to rely on additional uncharacterized molecular players.

## Conclusions

**Figure 9:**
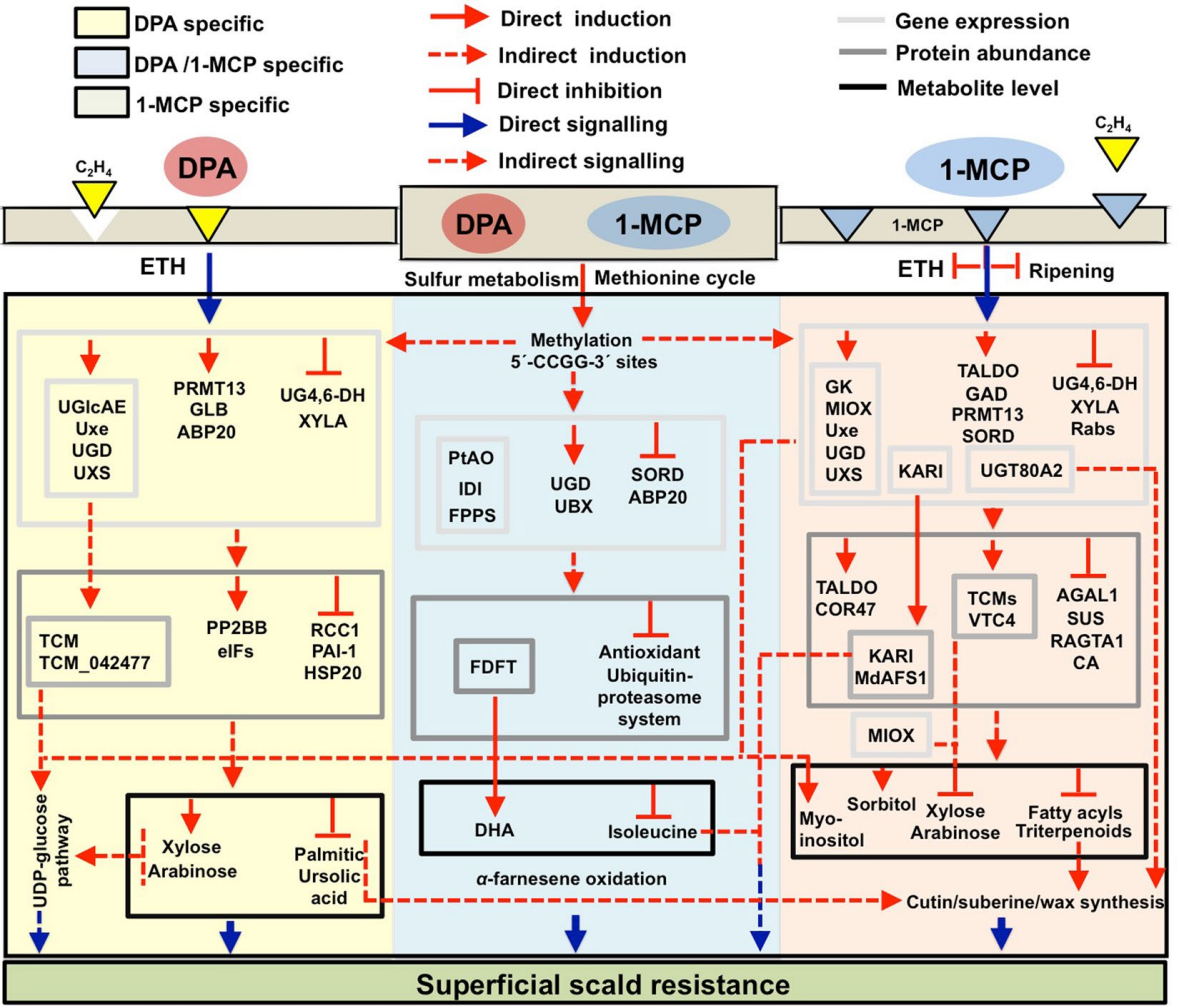
A model of how DPA and 1-MCP may induce scald resistance in ‘Granny Smith’ apple.

In summary, combined multi-omics analyses and pathway-oriented outputs in ‘Granny Smith’ apples with contrasting superficial scald phenotypes following treatments with DPA and 1-MCP present a robust resource to study ethylene–dependent and –independent scald etiology. We identified several genes, proteins and metabolites undergoing major changes at specific pro-symptomatic and symptomatic stages that might be used as novel biomarkers for delineating scald resistance. Although there was some similarity in the patterns of expression in apples exposed to DPA and 1-MCP, specific expression hallmarks were unique to each chemical treatment. Based on these results, we proposed a regulatory model (Fig. 9), which explains the scald resistance-related changes in ‘Granny Smith’ skin tissue. These results provide insights into the long-standing mystery of scald syndrome, thereby enabling our ability to control this physiological disorder.

## Material and Methods

### Fruit material and sampling strategy

Apples ‘Granny Smith’ (*Malus domestica* Borkh.) were harvested at physiologically mature stage (firmness: 7.61 ± 0.09 kg, soluble solids content: 11.4 ± 0.44%, titratable acidity (malic acid, %): 0.82 ± 0.01%, dry weight: 13.94 ± 0.51%, starch content: 9.29 ± 0.17 mg g^−1^ fresh weight) in a commercial orchard at Imathia (North Greece). Fruits were randomly divided into three (3) groups of 125 fruits each. Apples of the first group were treated with diphenylamine (DPA) (2000 ppm) for 1 min at room temperature (20 °C). The second group of fruits was treated with 1-MCP (SmartFresh^SM^, AgroFresh, USA) (1000 ppm) for 24 h at 0 °C. Immediately after treatments, apples of all 3 groups were cold stored (0 °C, 95% RH) for 3 months. Following cold storage, fruits were transferred to room temperature (20 °C) where apple samples were analyzed after 0, 2, 4 and 8 days (d). During sampling both pericarp (skin) and outer pericarp (flesh) samples were collected from each replication per sample (3 batches of tissue from 7 fruits) were frozen with liquid nitrogen and stored (−80 °C).

### Physiological analysis of apple ripening behavior

Fruit firmness was measured by penetration of the 2 opposite sites of each fruit after peeling (1 mm thick) of skin using a texture analyzer (model 53205, T.R. Turoni srl, Forlì, Italy) with a 11 mm probe. Soluble solids content (SSC) was assessed in juice using a refractometer (Atago PR-1, Atago Co Ltd., Tokyo, Japan) and titratable acidity (TA) by titration^66^.

Statistical analysis transacted using SPSS 20.0 (SPSS, Chicago, IL, USA). Data (means consisted of three biological replications of 7 fruits per replication) were subjected to analysis of variance and least significant differences (LSD) at 5% level were used for means comparison.

### Determination of ethylene and respiration rate

Ethylene production and respiration rate were measured at 0, 2, 4 and 8 d ripening. For each treatment 3 replications of 2 fruits were weighed and placed into separate 2 L volume airtight jars for 1 hour. Ethylene and CO_2_ determination were conducted by withdrawing a 1 mL headspace gas sample from each airtight jar and injecting it into a gas chromatograph (model GC-2014ATF Shimadzu), equipped with a stainless steel column (filled with Porapak P, Q, R, S), a flame ionization detector (FID) (for ethylene determination) and a TCD detector (for CO_2_ determination) as detailed^21^ Statistical analysis performed as described above.

### Evaluation of superficial scald symptoms

The percentage of superficial scald surface was recorded as incidence and severity on affected fruit using a scale where 0 = none, 1 = 1–10%, 2 = 11–33%, 3 = 34–66%, and 4 = 67–100% of the surface area affected^3^. Skin foreground and background color on two opposites, free of symptoms, sides of each fruit were measured objectively using a colorimeter (Konica Minolta CR200 Chroma Meter, Konica Minolta Sensing, Inc., Osaka, Japan) and the CIE (Commission Internationale de l′Eclairage) parameters (L*, a*, b*, Hue angle and Chroma). Statistical analysis performed as described above.

### Proteomic analysis (LC-MS/MS)

Skin tissue samples just after scald symptoms development (4 d at 20 °C) were processed as outlined before^66^. The protein pellets comprising two biological replicas of each conditions (control, DPA and 1-MCP) were resuspended in 8 M Urea/100 mM Tris-HCl pH 8.5 (UA), and the extracted proteins were processed according to the Filter Aided Sample Preparation (FASP) protocol (using spin filter devices with a 10 kDa cut-off (Sartorius, VN01H02). Proteins on the filters were extensively washed through centrifugation at 14.000xg with the UA solution, reduced with 100 mM DTT in UA for 30 min and subsequently alkylated with 10 mg mL^−1^ iodoacetamide in the urea solution for 30 min in the dark. Proteins on the top of the filters were washed three times with 50 mM ammonium bicarbonate and finally digested in a thermomixer overnight at 37 °C and with 300 rpm by adding 1 μg trypsin/LysC mix in 80 μL of 50 mM ammonium bicarbonate solution (Mass spec grade, Promega). Peptides were eluted by centrifugation and dried down completely by speed-vac-assisted solvent removal. Dried peptides eluates were reconstituted in 0,1% (v/v) formic acid and 2% (v/v) acetonitrile in water and subjected to 1 min sonicating water bath. Peptide concentration was determined by nanodrop absorbance measurement at 280 nm.

The raw files were analysed with MaxQuant (version 1.6.0.16) searching against the GDDH13_1-1_prot.fasta database (45.116 entries, downloaded at 02/10/2017 from the GDR). Search parameters included a molecular weight ranging from 350 to 5.000 Da, a precursor mass tolerance of 20 ppm, MS/MS fragment tolerance of 0.5 Da, a maximum of two missed cleavages by trypsin, and methionine oxidation, deamination of asparagine and glutamine and protein N-terminal acetylation were set as variable modifications. Carbamidomethylation was set as fixed cysteine modification. Label-free quantification (LFQ) was performed in MaxQuant. The protein and peptide false discovery rate (FDR) was set to 1%. The match-between-run function was enabled.

Statistical analyses and data visualization were performed using Perseus (version 1.6.0.2). Proteins identified as “potential contaminants”, “reverse” and “only identified by site” were filtered out. The LFQ intensities were transformed to logarithmic. Zero intensity was imputed i.e. replaced by normal distribution, assuming that the corresponding protein is present in low amounts in the sample. The three (3) biological replicas plus two (2) corresponding technical replicas were grouped for each treatment and a two-sided Welch’s T-test of the grouped proteins was performed using p value (<0.05) for truncation (Supplementary Table S1). Protein annotations were added from the text file downloaded from GDR site (Dated 02/10/2017). A detailed list of the proteins identified and quantified (as z-scores) as well as their available GDR annotations such as KEGG orthologs, interPro domains, GO, is provided in Supplementary Table S1. Moreover, an enrichment analysis based on GO terms, KEGG pathways as well as the functional classification of the statistically significant regulated proteins was conducted (Supplementary Table S6). The approach used, was the statistical Fisher exact test and the p value threshold was set to <0.02.

### Transcriptomic study by quantitative real-time PCR of targeted gene

RNA was isolated from skin after cold storage (0 d at 20 °C) and after scald symptoms developed (4 d at 20 °C) using the RNeasy plant RNA isolation kit (Qiagen, Crawley, UK). RT-PCR was performed according to^21^. Primer assays (see Supplemental Table S2) for the selected genes were designed with Primer3Plus (http://www.bioinformatics.nl/cgi-bin/primer3plus/primer3plus.cgi). Additionally, specificity of the assays was tested *in silico* using blast (http://blast.ncbi.nlm.nih.gov/Blast.cgi). The qPCR was performed according to^67^. The cycling program was: two min at 95 °C to activate the polymerase, followed by 35 cycles of denaturation at 95 °C for 10 s, annealing at 55 to 62 °C for 15 s and elongation at 72 °C for 20 s. Post-PCR melting curves were measured from 65 to 95 °C in 0.5 °C intervals to validate the formation of expected PCR products. Data were analyzed with GenEx (MultiD, version 6.1). Off-scale data were removed during pre-processing using a cutoff at 36 cycles and outliers were identified with Grubb’s test. All data were normalized to the spike and converted to relative quantities (relative to the highest Ct for each gene, after arbitrarily assigning an expression of one to the least-expressed sample). The last step of pre-processing was to transform the data to log_2_ scale.

### Methylation Sensitive Amplification Polymorphism (f-MSAP) analysis and data scoring

DNA extraction from skin tissues was performed using NucleoSpin Plant II Kit (Macherey Nagel, Duren, Germany), according to manufacturer’s instructions. For each sample, genomic DNA (200 ng) was double-digested with *EcoRI/HpaII* and *EcoRI/MspI* (New England, Biolabs), followed by ligation of two different adapters and a pre-amplification reaction. Diluted pre-amplified fragments were used as starting material for selective amplification with selected primers (Supplemental Table S3). Fragment separation and detection from selective amplification was performed using an ABI PRISM 3730xl DNA sequencer. Genemapper 4.0 (Applied Biosystems) was used for scoring MSAP fragments and provided a matrix where alleles are presented with “1” (presence of fragment) and with “0” (absence of fragment)^23^.

Data were analyzed employing the program MSAP analyzer (http://mirna.imbb.forth.gr/MSAPAnalyzer.html). This package proceeds with analysis of epigenetic variation starting from a binary matrix indicating the banding pattern between the isoschizomeric endonucleases *HpaII* and *MspI*. Every fragment was scored as either present or absent in both *EcoRI-HpaII* and *EcoRI-MspI* amplification products, epigenetic differentiation among treatments was assessed and different event types of methylation was calculated (*de novo* methylation, demethylation, no change and other variation).

### Polar and non-polar metabolites analysis

Extraction of primary polar and non-polar metabolites was performed after cold storage (0 d at 20 °C) and after scald symptoms developed (4 d at 20 °C), as described^68–71^, with slight modifications. In both cases, the dried residues were re-dissolved by gentle shaking in 40 μL of 20 mg mL^−1^ methoxyamine hydrochloride for 120 min at 37 °C and subsequently were treated with 70 μL of N-methyl-N-(trimethylsilyl) trifluoroacetamide reagent (MSTFA) and incubated for 30 min at 37 °C. Gas chromatography–mass spectrometry (GC-MS) analysis was carried out in Thermo Trace Ultra GC equipped with ISQ MS and TriPlus RSH autosampler (Switzerland). One microliter was injected with a split ratio of 70:1. GC separation was held on a TR-5MS capillary column 30 m × 0.25 mm × 0.25 μm. Injector temperature was 220 °C, ion source 230 °C and interface 250 °C. A constant flow rate of 1 mL min^−1^ was used for carrier gas. The GC temperature program was carried out for 5 min at 70 °C, then increased to 260 °C (rate 8 °C min^−1^), where it remained for 15 min. Mass range of m/z 50–600 was recorded, after 5 min of solvent delay. The mass spectra were acquired in electron impact ionization mode. The peak area integration and chromatogram visualization were performed using X-calibur processing program. Standards were used for peak identification or NIST11 and GOLM databases in case of unknown peaks. The detected metabolites were assessed based on their relative response compared to the internal standards of adonitol and nonadecanoic acid for polar and non-polar metabolites respectively, and expressed as relative abundances^72^. Numerical values are available in Supplementary Table S4. A complete list of the identified metabolites, including their chemical formula, molecular mass, classification, IUPAC name and the metabolic pathways involved are presented (Supplementary Table S5).

## Electronic supplementary material

Additional Information

Dataset 1

Dataset 2

Dataset 3

Dataset 4

Dataset 5

Dataset 6

## References

[1] Knight MR, Knight H (2012). Low-temperature perception leading to gene expression and cold tolerance in higher plants. New Phytologist.

[2] Zermiani M (2015). Ethylene negatively regulates transcript abundance of ROP-GAP rheostat-encoding genes and affects apoplastic reactive oxygen species homeostasis in epicarps of cold stored apple fruits. Journal of Experimental Botany.

[3] Watkins CB, Bramlage WJ, Cregoe B (1995). A. Superficial Scald of ‘Granny Smith’ Apples is Expressed as a Typical Chilling Injury. Journal of American Society of Horticultural Science.

[4] Lurie S, Watkins CB (2012). Superficial scald, its etiology and control. Postharvest Biology and Technology.

[5] Apollo Arquiza JMR, Hay AG, Nock JF, Watkins CB (2005). 1-Methylcyclopropene interactions with diphenylamine on diphenylamine degradation, alpha-farnesene and conjugated trienol concentrations, and polyphenol oxidase and peroxidase activities in apple fruit. Journal of Agricultural and Food Chemistry.

[6] Rupasinghe HPV, Paliyath G, Murr DP (2000). Sesquiterpene α-farnesene synthase: partial purification, characterization, and activity in relation to auperficial scald development in apples. Journal of American Society of Horticultural Science.

[7] Busatto N (2014). Target metabolite and gene transcription profiling during the development of superficial scald in apple (Malus x domestica Borkh). BMC Plant Biology.

[8] Rudell DR, Mattheis JP, Hertog MLATM (2009). Metabolomic change precedes apple superficial scald symptoms. Journal of Agricultural and Food Chemistry.

[9] Farneti B (2014). Untargeted metabolomics investigation of volatile compounds involved in the development of apple superficial scald by PTR-ToF-MS. Metabolomics.

[10] Busatto N (2018). Apple fruit superficial scald resistance mediated by ethylene inhibition is associated with diverse metabolic processes. Plant Journal.

[11] Du L, Song J, Campbell Palmer L, Fillmore S, Zhang ZQ (2017). Quantitative proteomic changes in development of superficial scald disorder and its response to diphenylamine and 1-MCP treatments in apple fruit. Postharvest Biology and Technology.

[12] Daccord N (2017). High-quality de novo assembly of the apple genome and methylome dynamics of early fruit development. Nature Genetics.

[13] Tassoni A, Watkins CB, Davies PJ (2006). Inhibition of the ethylene response by 1-MCP in tomato suggests that polyamines are not involved in delaying ripening, but may moderate the rate of ripening or over-ripening. Journal of Experimental Botany.

[14] Schrick K (2009). Mutations in UDP-Glucose: sterol glucosyltransferase in Arabidopsis cause transparent testa phenotype and suberization defect in seeds. Plant Physiology.

[15] Misra RC, Kamthan M, Kumar S, Ghosh S (2016). A thaumatin-like protein of *Ocimum basilicum* confers tolerance to fungal pathogen and abiotic stress in transgenic Arabidopsis. Scientific Reports.

[16] Mei X (2016). Dual mechanisms regulating and accumulation of gamma- aminobutyric acid in tea (*Camellia sinensis*) leaves exposed to multiple stresses. Scientific Reports.

[17] Grones P (2017). Auxin-binding pocket of ABP1 is crucial for its gain-of-function cellular and developmental roles. Journal of Experimental Botany.

[18] Slocum RD (2005). Genes, enzymes and regulation of arginine biosynthesis in plants. Plant Physiology and Biochemistry.

[19] Nosarzewski M, archbold DD (2017). Tissue-specific expression of *Sorbitol Dehydrogenase* in apple fruit during early development. Journal of Experimental.

[20] Wegrzyn T, Macrae EA, Redgwell RJ (1994). Apple β-Galactosidase. Plant Physiology.

[21] Tanou G (2017). Exploring priming responses involved in peach fruit acclimation to cold stress. Scientific Reports.

[22] Tsai W, Reineke LC, Jain A, Jung SY, Lloyd RE (2017). Histone arginine demethylase JMJD6 is linked to stress granule assembly through demethylation of the stress granule nucleating protein G3BP1. Journal of Biological Chemistry.

[23] Avramidou EV, Ganopoulos IV, Doulis AG, Tsaftaris AS, Aravanopoulos FA (2015). Beyond population genetics: natural epigenetic variation in wild cherry (Prunus avium). Tree Genetics & Genomes.

[24] Tsantili E (2007). Ethylene and α-Farnesene Metabolism in Green and Red Skin of Three Apple Cultivars in Response to 1-Methylcyclopropene (1-MCP) Treatment. Journal of Agricultural and Food Chemistry.

[25] Yao YX, Dong QL, Zhai H, You CX, Hao YJ (2011). The functions of an apple cytosolic malate dehydrogenase gene in growth and tolerance to cold and salt stresses. Plant Physiology and Biochemistry.

[26] Trobacher CP (2013). Calmodulin-dependent and calmodulin-independent glutamate decarboxylases in apple fruit. BMC Plant Biology.

[27] Yang X (2013). Effect of ethylene and 1-MCP on expression of genes involved in ethylene biosynthesis and perception during ripening of apple fruit. Postharvest Biology and Technology.

[28] Ranwala AP, Suematsu C, Masuda H (1992). The Role of beta-Galactosidases in the modification of cell wall components during muskmelon fruit ripening. Plant Physiology.

[29] Wang Q (2012). The γ-carbonic anhydrase subcomplex of mitochondrial complex I is essential for development and important for photomorphogenesis of Arabidopsis. Plant Physiology.

[30] Caillau M, Quick WP (2005). New insights into plant transaldolase. Plant Journal.

[31] Griffith M (2007). Thellungiella: an Arabidopsis -related model plant adapted to cold temperatures. Plant, Cell and Environment.

[32] Abbal P (2008). Molecular characterization and expression analysis of the Rab GTPase family in Vitis vinifera reveal the specific expression of a VvRabA protein. Journal of Experimental.

[33] Woollard AAD, Moore I (2008). The functions of Rab GTPases in plant membrane traffic. Current Opinion in Plant Biology.

[34] Rutherford S, Moore I (2002). The *Arabidopsis* Rab GTPase family: another enigma variation. Current Opinion in Plant Biology.

[35] The Arabidopsis Genome Iniative. Analysis of the genome sequence of the flowering plant *Arabidopsis thaliana*. *Nature* **408**, 796–815 (2000). 10.1038/35048692 11130711

[36] Echevarría-zomeño S, Yángüez E, Fernández-bautista N (2013). Regulation of Translation Initiation under Biotic and Abiotic Stresses. International Journal of Molecular Sciences.

[37] Ziogas V (2015). Roles of sodium hydrosulfide and sodium nitroprusside as priming molecules during drought acclimation in citrus plants. Plant Molecular Biology.

[38] Wang X, Cai X, Xu C, Wang Q, Dai S (2016). Drought-responsive mechanisms in plant leaves revealed by proteomics. International Journal of Molecular Sciences.

[39] Ravanel S, Gakière B, Job D, Douce R (1998). The specific features of methionine biosynthesis and metabolism in plants. Proceedings of the National Academy of Sciences of United States of America.

[40] Saito K (2000). Regulation of sulfate transport and synthesis of sulfur-containing amino acids. Current Opinion in Plant Biology.

[41] Galili G, Amir R, Hoefgen R, Hesse H (2005). Improving the levels of essential amino acids and sulfur metabolites in plants. Biological Chemistry.

[42] Curien G, Job D, Douce R, Dumas R (1998). Allosteric activation of *Arabidopsis* threonine synthase by S-adenosylmethionine. Biochemistry.

[43] Alban C, Job D, Douce R (2000). Biotin metabolism in plants. Annual Review of Plant Physiology and Plant Molecular Biology.

[44] Oge, L. *et al*. Protein repair L-isoaspartyl methyltransferase1 is involved in both seed longevity and germination vigor in *Arabidopsis*. *Plant Cell* **20**, 3022–3037 (2008). 10.1105/tpc.108.058479 PMC2613667 19011119

[45] Farinati S, Rasori A, Varotto S, Bonghi C (2017). Rosaceae fruit development, ripening and post-harvest: an epigenetic rerspective. Frontiers in Plant Science.

[46] Gapper NE, Rudell DR, Giovannoni JJ, Watkins CB (2017). Biomarker development for external CO_2_ injury prediction in apples through exploration of both transcriptome and DNA methylation changes. Annals of Botany.

[47] Campos ACE (2007). Oxidative stress modulates DNA methylation during melanocyte anchorage blockade associated with malignant Transformation. Neoplasia.

[48] Groth M (2016). MTHFD1 controls DNA methylation in *Arabidopsis*. *Nature*. Communications.

[49] Van de Poel B (2013). S-adenosyl-L-methionine usage during climacteric ripening of tomato in relation to ethylene and polyamine biosynthesis and transmethylation capacity. Physiologia Plantarum.

[50] Feng X, An Y, Zheng J, Sun M, Wang L (2016). Proteomics and SSH analyses of ALA-Promoted fruit coloration and evidence for the involvement of a MADS-BoxGene, MdMADS1. Frontiers in Plant Science.

[51] Gutensohn M (2013). Cytosolic monoterpene biosynthesis is supported by plastid-generated geranyl diphosphate substrate in transgenic tomato fruits. Plant Journal.

[52] Zou J (2018). Transcriptome analysis of aroma volatile metabolism change in tomato (*Solanum lycopersicum*) fruit under different storage temperatures and 1-MCP treatment. Postharvest Biology and Technology.

[53] Phillips MA, Croteau RB (1999). Resin-based defenses in conifers. Trends in Plant Science.

[54] Lee J, Rudell DR, Watkins CB (2014). Metabolic changes in 1-methylcyclopropene (1-MCP)-treated ‘Empire’ apple at different storage temperatures. Acta Horticulturae.

[55] Joshi V, Joung JG, Fei Z, Jander G (2010). Interdependence of threonine, methionine and isoleucine metabolism in plants: accumulation and transcriptional regulation under abiotic stress. Amino Acids.

[56] Goddard NJ (1993). Molecular analysis and spatial expression pattern of a low-temperature-specific barley gene, blt101. Plant Molecular Biology.

[57] Matich AJ, Banks NH, Rowan DD (1998). Modification of  *α*-farnesene levels in cool-stored ‘Granny Smith’ apples by ventilation. Postharvest Biology and Technology.

[58] Moggia C, Moya-Leon M, Pereira M, Yuri J, Lobos G (2010). Effect of DPA and 1-MCP on chemical compounds related to superficial scald of Granny Smith apples. Spanish Journal of Agricultural Research.

[59] Latimer SB (1964). Fatty-acid accumulation by acrylate inhibition of β-oxidation in an alkane- oxidizing Pseudomonas. Biochimica et Biophysica Acta.

[60] Lara I, Belge B, Goulao LF (2015). A focus on the biosynthesis and composition of cuticle in fruits. Journal of Agriculture and Food Chemistry.

[61] Bain J, Mercer FV (1963). The submicroscopio cytology of superficial scald, a physiological disease of apples. Australian Journal of Biological Sciences.

[62] Rautengarten C, Birdseye D, Pattathil S, Mcfarlane HE, Saez-aguayo S (2017). The elaborate route for UDP-arabinose delivery into the Golgi of plants. Proceedings of the National Academy of Sciences.

[63] Tenhaken R (2015). Cell wall remodeling under abiotic stress. Frontiers in Plant Science.

[64] Ahn J (2006). Depletion of UDP-D-apiose/UDP-D-xylose synthases results in rhamnogalacturonan-II deficiency, cell wall thickening, and cell death in higher plants. Journal of Biological Chemistry.

[65] Gapper NE (2017). Delayed response to cold stress is characterized by successive metabolic shifts culminating in apple fruit peel necrosis. BMC Plant Biology.

[66] Karagiannis E (2016). Comparative Physiological and Proteomic Analysis Reveal Distinct Regulation of Peach Skin Quality Traits by Altitude. Frontiers in Plant Science.

[67] Xanthopoulou A (2017). De novo comparative transcriptome analysis of genes involved in fruit morphology of pumpkin cultivars with extreme size difference and development of EST-SSR markers. Gene.

[68] Kim HK, Verpoorte R (2010). Sample preparation for plant metabolomics. Phytochemical Analysis.

[69] O’Gorman A, Barry-Ryan C, Frias JM (2012). Evaluation and identification of markers of damage in mushrooms (*Agaricus bisporus*) postharvest using a GC/MS metabolic profiling approach. Metabolomics.

[70] Michailidis M (2017). Metabolomic and physico-chemical approach unravel dynamic regulation of calcium in sweet cherry fruit physiology. Plant Physiology and Biochemistry.

[71] Karagiannis E (2018). Postharvest responses of sweet cherry fruit and stem tissues revealed by metabolomic profiling. Plant Physiology and Biochemistry.

[72] Ainalidou A (2015). Integrated analysis of metabolites and proteins reveal aspects of the tissue-specific function of synthetic cytokinin in kiwifruit development and ripening. Journal of Proteomics.

[73] Bevan M (1998). Analysis of 1.9 Mb of contiguous sequence from chromosome 4 of *Arabidopsis thaliana*. Nature.

