# Supplementary material for: Ethylene –dependent and –independent superficial scald resistance mechanisms in ‘Granny Smith’ apple fruit
Source: Sci Rep. 2018 Jul 30;8:11436. doi: 10.1038/s41598-018-29706-x (PMC6065312; doi:10.1038/s41598-018-29706-x)
Supplement: Supplementary file 1 — Additional Information [file 41598_2018_29706_MOESM1_ESM.pdf]

Ethylene –dependent and –independent superficial scald resistance mechanisms in 'Granny Smith' apple fruit

Evangelos Karagiannis, Michail Michailidis, Georgia Tanou, Martina Samiotaki, Katerina Karamanoli, Evangelia Avramidou, Ioannis Ganopoulos, Panagiotis Madesis, Athanassios Molassiotis

Supplementary data

Supplementary Figures 1-2 and Supplementary Tables 1-6

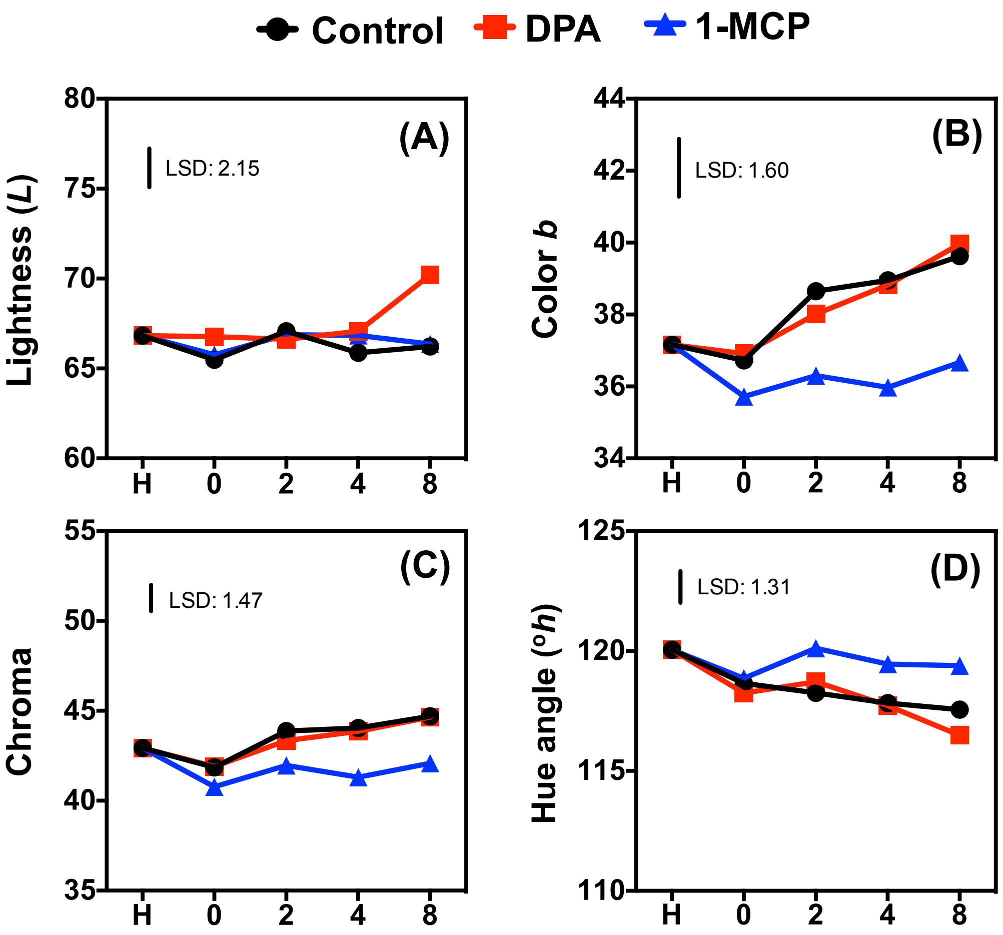

Supplementary Figure 1

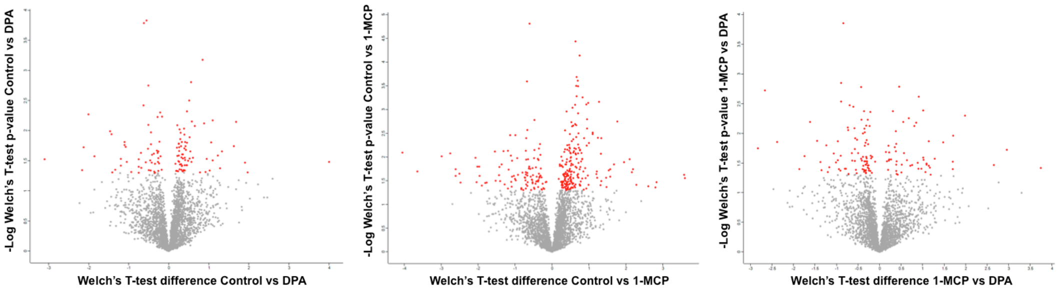

Supplementary Figure 2

Additional information

Supplementary Fig. S1. *Color changes* throughout apple *ripening*.

Supplementary Fig. S2. *Volcano plots generated for each performed comparison, using the Welch's T-test results: Control versus DPA (A), Control versus 1-MCP (B), DPA versus 1-MCP (C). Protein spots in red are statistically significant (< 0.01) altered and their identification details can be found in Supplementary Table S1.*

Supplementary Table S1. Protein identification of apple skin following label free-gel proteomic analysis.

Supplementary Table S2. Sequences of *primers used for* RT-PCR.

Supplementary Table S3. Sequences of adapters for the *EcoRI* and *HpaII/MspI* ligation step, and for the pre-selective (*EcoRI*+1, *HpaII/MspI*+1) and selective (*EcoRI*+3, HpaII/*MspI*+3) amplifications.

Supplementary Table S4. Apple skin metabolites that affected by DPA and 1-MCP.

Supplementary Table S5. Details of identified metabolites and their participation in biological pathways.

Supplementary Table S6: Enrichment analysis based on significance according to Fisher exact test (p-value)
